# The Gut Microbiota in Immune-Mediated Inflammatory Diseases

**DOI:** 10.3389/fmicb.2016.01081

**Published:** 2016-07-11

**Authors:** Jessica D. Forbes, Gary Van Domselaar, Charles N. Bernstein

**Affiliations:** ^1^Department of Medical Microbiology and Infectious Diseases, University of Manitoba, WinnipegMB, Canada; ^2^National Microbiology Laboratory, Public Health Agency of Canada, WinnipegMB, Canada; ^3^Department of Internal Medicine and the IBD Clinical and Research Centre, University of Manitoba, WinnipegMB, Canada

**Keywords:** microbiome, systems microbiology, chronic immune mediated inflammatory diseases, metagenome, dysbiosis

## Abstract

The collection of microbes and their genes that exist within and on the human body, collectively known as the microbiome has emerged as a principal factor in human health and disease. Humans and microbes have established a symbiotic association over time, and perturbations in this association have been linked to several immune-mediated inflammatory diseases (IMID) including inflammatory bowel disease, rheumatoid arthritis, and multiple sclerosis. IMID is a term used to describe a group of chronic, highly disabling diseases that affect different organ systems. Though a cornerstone commonality between IMID is the idiopathic nature of disease, a considerable portion of their pathobiology overlaps including epidemiological co-occurrence, genetic susceptibility loci and environmental risk factors. At present, it is clear that persons with an IMID are at an increased risk for developing comorbidities, including additional IMID. Advancements in sequencing technologies and a parallel explosion of 16S rDNA and metagenomics community profiling studies have allowed for the characterization of microbiomes throughout the human body including the gut, in a myriad of human diseases and in health. The main challenge now is to determine if alterations of gut flora are common between IMID or, if particular changes in the gut community are in fact specific to a single disease. Herein, we review and discuss the relationships between the gut microbiota and IMID.

## Introduction

Immune-mediated inflammatory disease (IMID) is a term used to define a group of clinically heterogeneous, ostensibly unrelated disorders that are recognized to share common pathogenic mechanisms. Most IMID are highly prevalent in well-developed industrialized countries; in Western populations the prevalence of IMID is approximately 5–8% (Bayry and Radstake, 2013) and encompasses over 100 different clinical diseases including inflammatory bowel disease (IBD), multiple sclerosis (MS), rheumatoid arthritis (RA), ankylosing spondylitis (AS), systemic lupus erythematosus (SLE), and psoriasis/psoriatic arthritis.

Knowledge of the etiopathogenic mechanisms of IMID remains limited but these diseases are thought to arise secondary to a complex interplay between environmental and genetic factors. The rise in IMID prevalence in developed countries in the latter half of the 20th century implies that environmental determinants play a significant role in disease onset. Numerous environmental factors have been proposed to be important in the emergence of IMID including hygiene, socioeconomic status, cigarette smoking, diet, antibiotic usage, vitamin D, hormones, appendectomy, excess alcohol, and microbial exposure (Ananthakrishnan, 2015; Belbasis et al., 2015), but whether any of these factors are causal in IMID has yet to be established.

Immune-mediated inflammatory disease also share a considerable portion of their heritable etiology. This is evident through familial clustering of multiple IMID, epidemiological co-occurrence and a similar efficacy of therapeutics directed at specific biologic loci across some diseases (Somers et al., 2006; Diaz-Gallo and Martin, 2012), which taken together, suggest that genetic factors predispose persons to IMID. The best-known genetic factor of IMID is the human leukocyte antigen (HLA) haplotypes (Wu et al., 2015). Moreover, genome-wide association studies have identified associations with more than 200 rare or common non-HLA variants (Wu et al., 2015) although known allelic variants account for a relatively low risk explaining 20–50% of disease heritability (Parkes et al., 2013; Sorrentino, 2014).

## Immune-Mediated Inflammatory Disease Comorbidity

Immune-mediated inflammatory disease are defined by the principle organ system that is affected [the gastrointestinal tract in IBD, the synovium in RA and the central nervous system (CNS) in MS]; however, they are associated with comorbidities that extend beyond the primary target organ. In this regard, comorbidities that present in persons with IMID greatly contribute to the burden of disease and quality of life. IBD for example, frequently manifests with extra-intestinal complications in up to 50% of cases and covers a broad clinical spectrum affecting nearly every organ system (Harbord et al., 2016). Erythema nodosum and pyoderma gangrenosum of skin, ocular uveitis and episcleritis, primary sclerosing cholangitis, and arthritides, such as pauciarticular and polyarticular peripheral arthritis and seronegative spondyloarthropathy are among the more common extra-intestinal diseases that may coexist in IBD (Harbord et al., 2016). Rarely, a number of neurological complications including peripheral neuropathy, myopathy, and demyelinating disorders may occur in persons with IBD. Comorbidities are not specific to IBD; persons with other IMID often present with a number of additional clinical disorders including risks for osteoporosis, venous thromboembolic disease and ischemic heart disease (Bernstein et al., 2000, 2001a, 2008).

It is well established that in comparison to the general population, IMID patients are at greater risk for the development of another IMID-related condition. Weng et al. (2007) examined the co-occurrence of IBD with other IMID (asthma, psoriasis, type 1 diabetes (T1D), RA, MS, SLE, vitiligo, autoimmune thyroiditis, and chronic glomerulonephritis). The authors reported 17% of IBD patients and 10% of persons without IBD were diagnosed with at least one additional IMID. Persons with IBD had significantly increased odds pertaining to the development of asthma [odds ratio; OR 1.5, 95% confidence interval (CI) 1.4–1.6], psoriasis (OR 1.7, 95% CI 1.5–2.0), RA (OR 1.9, 95% CI 1.5–2.3), and MS (OR 2.3 95% CI 1.6–3.3). These results have been corroborated by other studies that add further credence to the validity of the phenomenon of IMID co-occurrence (Bernstein et al., 2001b, 2005; Cucino and Sonnenberg, 2001; Cohen et al., 2008; Marin-Jiminez et al., 2014; Vanaclocha et al., 2015).

An idiopathic etiology represents a pivotal commonality of IMID, though the clinical, epidemiological, genetic, and environmental links between IMID combined with the preeminent association between IBD and the gut microbiome has laid the foundation for studies evaluating the gut microbiome in IMID. Intriguingly, recent research suggests there are striking findings to support the gut microbiome as playing an important function in the etiopathogenesis of several IMID. Herein, we will review the current state of the knowledge regarding changes in the gut microbiome of the more common IMID, including IBD, MS, RA, AS, SLE, psoriasis, and psoriatic arthritis.

## Gut Microbiome: An Overview

Knowledge of the role of commensal microbes that inhabit mucosal surfaces of the human body and their role in health and disease is increasing at a remarkable rate. The term “microbiota” refers to the population of microbes at a particular anatomical niche and “microbiome” refers to the collective genes encoded by all microbes of that particular niche. The human gastrointestinal tract comprises approximately 10^14^ bacterial microbes and amounts to a biomass of approximately 2 kg (Qin et al., 2010). It has been assumed for some time that there are roughly 10 times as many microbes as there are eukaryotic cells in the human body, but recent support proposes the ratio for microbial to human cells is 1.3:1 (Abbott, 2016). The bacteria of the gut belong to more than 1000 different species encoding more than 3 million bacterial genes (microbiome) exceeding the number encoded by the human genome by 150-fold (Qin et al., 2010).

The study of the gut microbiome cannot be separated from its environmental context; host genetics, nutrition, the environment, geographical location, early microbial exposures, and other factors profoundly impact the microbiome of the healthy human gut. Extensive sampling of the human microbiome has revealed that although the diversity and abundance of microbial communities of particular niches vary widely both within and among individuals (Huttenhower et al., 2012), the functional repertoire of the healthy microbiome, including the gut, is relatively stable regardless of the microbe composition. A microbiome becomes more stable in adulthood, which subsequently changes in the elderly (Claesson et al., 2012). However, there are temporal variations within a healthy individual’s microbiome that can occur even over a short period of time (David et al., 2014).

The healthy human gut is dominated by the presence of four bacterial phyla: Bacteroidetes, Firmicutes, Actinobacteria, and Proteobacteria with Bacteroidetes and Firmicutes accounting for a large majority of endemic bacteria in the gut (Tap et al., 2009). Within the healthy human gut, the phylum Firmicutes are divided into two major classes of Gram-positive bacteria: Bacilli and Clostridia (primarily *Clostridium* cluster IV and *Clostridium* XIVa). The Bacteroidetes are Gram-negative bacteria, of which the *Bacteroides* represents one of the most abundant genera in the gut (Huttenhower et al., 2012). Considerable diversity of bacterial species exist within a normal gut; recent studies have recognized common core subsets within the microbiome that are relatively stable throughout large populations and that can even persist in an individuals’ gut for their entire adult life (Faith et al., 2013).

There is a clear spatial distribution of microbes within the gastrointestinal tract with diversity increasing from the stomach to the colon, and it is at the terminal ileum where prevalent species change from aerobes to anaerobes (Mondot et al., 2013). Within the gut itself, there is a significant difference in microbial populations on mucosal surfaces compared to within the lumen (Li et al., 2015). Microbes at the mucosal surface are in closer proximity to the intestinal epithelium and may have a greater influence on the immune system whereas luminal/fecal microbes might be more essential for energy and metabolic interactions. This is relevant since many studies of the gut microbiota use fecal material for community profiling and thus may not adequately reflect the totality of viable microbes within the gut.

Under normal physiological conditions, the human gut microbiota is a homeostatic ecosystem with several vital functions and interrelationships important to host health including food digestion, development of the host immune system and intestinal epithelial barrier and protection against pathogens (Shreiner et al., 2015). Disruption of this equilibrium can result in dysbiosis and increase risk of disease. Gut dysbiosis refers to an altered composition of intestinal microbial populations and is thought to provide continuous immunological stimulation leading to immune response anomalies in numerous IMID. Altered community composition has been established in a number of gastrointestinal diseases: IBD, celiac disease, irritable bowel syndrome, functional dyspepsia, antibiotic-associated diarrhea, tropical enteropathy, and others (Brown et al., 2015; Keely et al., 2015; D’Argenio et al., 2016; Distrutti et al., 2016; Larcombe et al., 2016). Accumulating evidence proposes that dysbiosis of the intestinal microbiota is not limited to gastrointestinal diseases thereby suggesting that gut bacteria can affect the systemic immunological response. A number of studies have investigated gut dysbiosis in relation to obesity, diabetes, chronic periodontitis, vaginosis, atopic diseases, non-alcoholic steatohepatitis, Alzheimer’s disease, and others (Daulatzai, 2014; Adams and Morrison, 2016; Blasco-baque et al., 2016; Johnson and Ownby, 2016). While a breakdown in the equilibrium of the intestinal milieu may be widely recognized, perturbations surrounding biological mechanisms driving dysbiosis exist and it is unclear whether dysbiosis manifests as a cause or consequence of disease.

## Microbiome and Immunity Link

**FIGURE 1:**
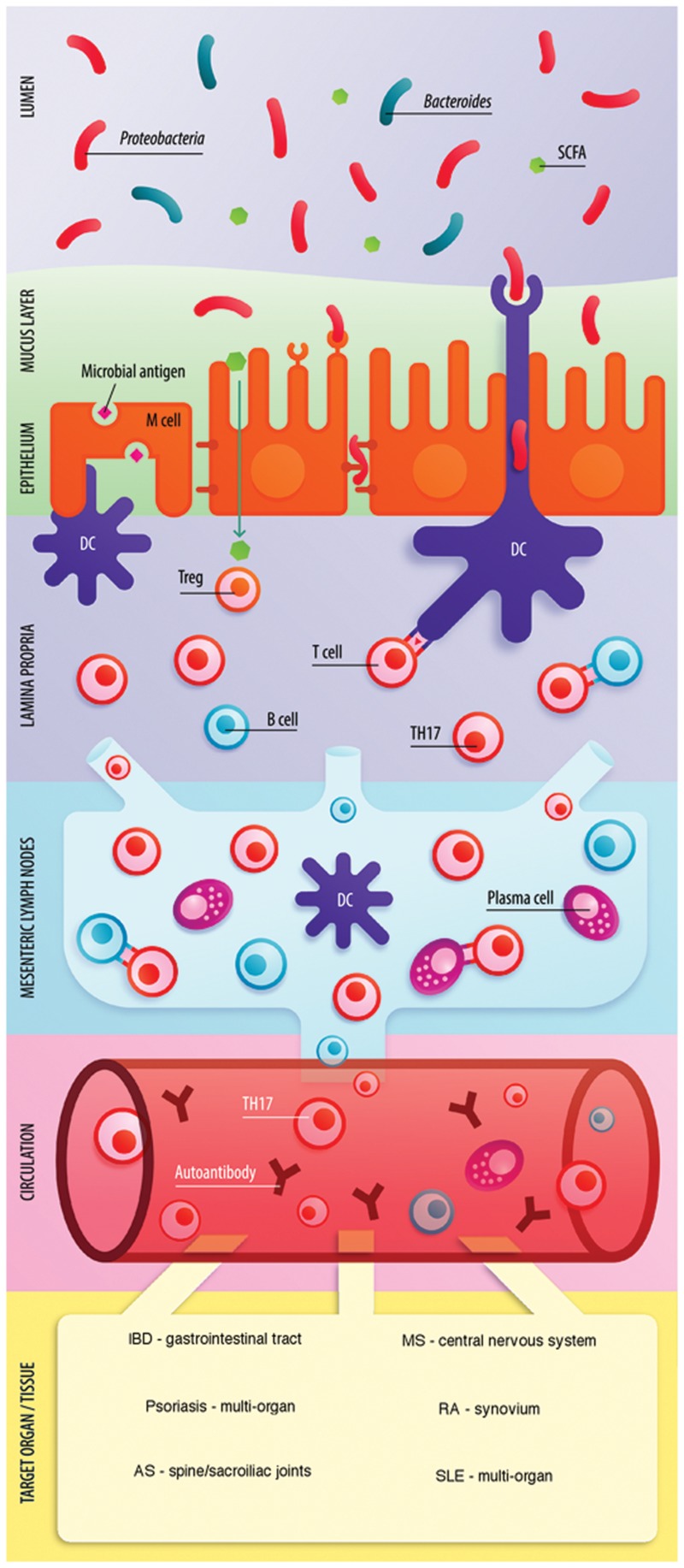
**Role of the gut microbiome in the pathogenesis of IMID.** Enterocytes (via recognition of microbe-associated molecular patterns by Toll-like receptors) and M cells (transcytosis) communicate microbial dysbiosis to the mucosal immune system. Gut dysbiosis, in combination with a reduction of SCFA can shift the Treg-Th17 balance toward inflammation through Th17 expansion. Several pro-inflammatory cytokines are released in the lamina propria (LP) including IL-6, IL-17, IL-21, IL-23, and IFN-γ. The inflammatory state enhances intestinal permeability through expression of tight junction proteins including occludins and claudins and may lead to microbial translocation and enhanced antigen exposure in the LP. Activated T cells, B cells and dendritic cells (DC) travel to the mesenteric lymph nodes (MLN) via lymphatic vessels. Antigen presentation by DCs to T cells occurs and B cells differentiate toward plasma cells. Activated B cells, Th17 cells, and antibody-producing plasma cells enter circulation. B cells may be stimulated to differentiate toward autoantibody producing plasma cells if exposed to mimicry antigen in LP or MLN. Increased Th17 cells and presence of autoantibodies are the result of the microbiota induced pro-inflammatory state. Figure adapted from van der Meulen et al. (2016) with permission and modified.

The link between the gut microbiome and host immunity involves a bidirectional relationship between microbes and the host innate and adaptive immune system. The balance between pro- and anti-inflammatory mechanisms is critical for gut immune homeostasis and is directly affected by the commensal microbial communities of the gut. For example, in mice, segmented filamentous bacteria (SFB) promote the accumulation of pro-inflammatory T helper 1 (Th1) and Th17 cells in the small intestine (Gaboriau-Routhiau et al., 2009). Anti-inflammatory responses are facilitated by the generation of regulatory T cells (Treg cells) through short-chain fatty acid (SCFA) production (Arpaia et al., 2013). Similarly, studies report retinoic acid (Mucida et al., 2007), bacteria belonging to the *Clostridium* cluster IV and XIVa (Atarashi et al., 2013), polysaccharide A of *Bacteroides fragilis* (Round and Mazmanian, 2010) and *Faecalibacterium prausnitzii* (Qiu et al., 2013) to promote the accumulation of Treg cells. Hence, microbes and metabolites in the gut are essential in maintaining immunological equilibrium. And it is suspected that perturbations of the gut microbiota disrupt this balance primarily affecting the gut mucosa and also the systemic immune response. A ‘leaky gut’ characterized by increased gut permeability, microbial imbalance, and impaired mucosal immunity has been identified to precede the development of IMID. **Figure 1** shows a schematic illustration of the interaction between the immune system and gut microbiota in IMID.

## Gut Microbiota in Immune-Mediated Inflammatory Diseases

### Inflammatory Bowel Disease (IBD)

The two main clinical phenotypes of IBD are Crohn’s disease (CD) and ulcerative colitis (UC). CD is a transmural disease characterized by deep ulcerations distributed in a skipped pattern that can affect the gastrointestinal tract anywhere from the mouth to the anus, though the terminal ileum is most frequently affected. In contrast, UC demonstrates continuous, superficial inflammation (of the mucosa and submucosa) involving the rectum and extending proximally to the cecum.

At present, not only have numerous candidate pathogens (adherent invasive *E. coli* (AIEC), *Bacteroides fragilis, Clostridium difficile, Mycobacterium avium* ssp. *paratuberculosis, Yersinia enterocolitica, Listeria monocytogenes, Salmonella, Campylobacter*) been implicated in disease onset and/or perpetuation (Becker et al., 2015), but over 400 complex microbiome surveys have been conducted to identify the totality of microbes in the IBD gut and precisely how the IBD gut differs from that of a healthy person. Indeed, advances in culture-independent sequencing and bioinformatics analyses in recent years have provided tremendous insight into the structure and function of the gut microbiota. Though patterns of gut dysbiosis are inconsistent among published studies, partly owing to specimen type (stool versus biopsy) or analysis methods, studies surveying the gut microbiome in persons with IBD have consistently demonstrated perturbations of the structure and function of the microbiome.

Studies frequently document an overall reduction of diversity, the total number of species in a community. In fact, data from the MetaHIT consortium suggest that persons with IBD harbor on average 25% fewer microbial genes than healthy persons (Qin et al., 2010). Diversity is reduced in the fecal and mucosal microbiomes of IBD (Manichanh et al., 2006; Lepage et al., 2011) and has also been documented among monozygotic twins discordant for CD (Dicksved et al., 2008). Decreased diversity has been attributed to shifts in the abundance of the Firmicutes, and more specifically the *Clostridium leptum* and *C. coccoides* group (Manichanh et al., 2006). Likewise, utilizing a custom phylogenetic microarray Kang et al. (2010) reported some bacteria belonging to the Firmicutes phylum including *Eubacterium rectale* of the Lachnospiraceae and *Ruminococcus albus, R. callidus, R. bromii*, and *F. prausnitzii* of the Ruminococcaceae were 5- to 10-fold more abundant in healthy persons compared to CD.

The gut microbiome was recently characterized according to the three most dominant phenotypes of CD: inflammation of the colon, the terminal ileum, or both (**Table 1**) (Naftali et al., 2016). The authors demonstrated ileal and colonic CD sustain distinct microbiome patterns. For example, ileal CD samples were richer in *Escherichia* (Enterobacteriaceae) and disease activity correlated with *Fusobacterium* abundance whereas colonic CD had higher levels of *Faecalibacterium* and two unidentified genera of the Clostridiales and Ruminococcaceae. The variance in CD microbiome patterns suggests that different mechanisms might underlie the two major clinical manifestations of CD. Recently, de Meij et al. (2015) described the gut microbiome composition in active new-onset pediatric CD compared with 3 months following initiation of treatment. At baseline, a greater diversity of Proteobacteria and a lower abundance of Bacteroidetes were observed in CD compared to controls. And upon clinical remission, the microbiota profile of CD seemed to shift to a profile similar to the control population.

**Table 1:** Current studies investigating the role of the gut microbiota in IMID patients.

IMID	Subjects	Comments	Reference
IBD	Thirty-one patients with ileal, ileocolic, and colon-restricted CD	Distinct microbiota profiles observed between ileal and colonic CD: ileal CD richer in *Escherichia* and disease activity correlated with abundance of *Fusobacterium*; increased abundance of *Faecalibacterium*, Clostridiales, and Ruminococcaceae in colonic CD.	Naftali et al., 2016
	
	Sixty newly diagnosed pediatric CD	Increased diversity of Proteobacteria and decreased abundance of Bacteroidetes in CD at baseline. Microbiota profile of CD resembled profile of controls upon clinical remission.	de Meij et al., 2015
	
	Four hundred and sixty-eight newly diagnosed pediatric CD	Increased abundance of Enterobacteriaceae, Pasteurellaceae, Veillonellaceae, Fusobacteriaceae and decreased abundance of Erysipelotrichales, Bacteroidales, and Clostridiales correlates with disease status. Shifts more strongly observed in tissue versus stool.	Gevers et al., 2014
	
	Fifteen patients with CD, 21 patients with UC	Bacteroidetes and Fusobacteria more abundant in inflamed CD mucosa versus inflamed UC. Proteobacteria and Firmicutes more abundant in inflamed UC mucosa. *Faecalibacterium, Bacteroides*, and *Pseudomonas* levels differ between inflamed CD and UC; 13 genera significantly differed within the non-inflamed mucosa. Non-inflamed UC mucosa most different from non-IBD mucosa. No variation in taxa when comparing different gut comparatments within CD or UC.	Forbes et al., 2016

MS	Seven RRMS patients	Differences in Firmicutes, Bacteroidetes, and Proteobacteria. *Faecalibacterium* decreased in MS. Differences in Bacteroidaceae, *Faecalibacterium, Ruminococcus*, Lactobacillaceae, *Clostridium*, and other Clostridiales in glatiramer acetate-treated patients compared to untreated. Vitamin D associated with changes in Firmicutes, Actinobacteria, and Proteobacteria. Untreated patients had increase in *Akkermansia, Faecalibacterium*, and *Coprococcus* after vitamin D treatment.	Mowry et al., 2012 Cantarel et al., 2015
	
	Fifty-three MS patients: 22 untreated; 13 glatiramer acetate treated; 18 IFN-β treated	Increased *Methanobrevibacter smithii* in MS. Decreased *Butyricimonas* and Lachnospiraceae in untreated MS.	Jhangi et al., 2014
	
	Twenty pediatric RRMS	Increased *Escherichia, Shigella, Clostridium*. Decreased *Eubacterium rectale, Corynebacterium*.	Tremlett et al., 2015
	
	Fifteen pediatric RRMS	IL-17^+^ T cells positively correlated with overall richness and evenness; inverse correlation between IL-17^+^ T cells and Bacteroidetes	Tremlett et al., 2016a
	
	Seventeen pediatric RRMS	Decreased Fusobacteria, increased Firmicutes and Euryarchaeota linked to shorter relapse time.	Tremlett et al., 2016b

RA	Fifty-one early (≤6 mo) RA	Decreased bifidobacteria, *Bacteroides fragilis* subgroup, *Eubacterium rectale – Clostridium coccoides* subgroup, and *Bacteroides–Porphyromonas–Prevotella* groups	Vaahtovuo et al., 2008
	
	Fifteen early (≤6 mo) RA	Increased *Lactobacillus* and diversity.	Liu et al., 2013
	
	Forty-four new-onset RA; 26 chronic, treated RA; 16 PsA	Increased *Prevotella copri* in NORA; decreased *Bacteroides* and beneficial microbes.	Sher et al., 2013
	
	Seventy-seven treatment naïve RA; 17 treatment naïve RA paired with healthy relatives; 21 DMARD-treated RA	Increased *Lactobacillus salivarius*; decreased *Haemophilus*. Metagenomic linkage groups containing *Clostridium asparagiforme, Gordonibacter pamelaeae, Eggerthella lenta, Lachnospiraceae, Bifidobacterium dentium, Lactobacillus* sp., and *Ruminococcus lactaris* enriched.	Zhang et al., 2015
	
	Forty RA	Expansion of Actinobacteria (*Eggerthella, Actinomyces*), *Turicibacter, Streptococcus*. Reduced *Faecalibacterium*. *Collinsella* correlated with high levels of alpha-aminoadipic acid and asparagine and IL-17A production. Role of *Collinsella* in altering gut permeability and disease severity confirmed in experimental arthritis.	Chen et al., 2016

AS	Fifteen AS patients	Increased prevalence of sulfate-reducing bacteria. Decreased immunological tolerance to *Bacteroides*.	Stebbings et al., 2002
	
	Nine recent-onset (≤48 mo) TNF-inhibitor naïve AS patients	Increased diversity, Lachnospiraceae, Ruminococcaceae, Rikenellaceae, Porphyromonadaceae, and Bacteroidaceae; decreased Veillonellaceae and Prevotellaceae	Costello et al., 2015

SLE	Twenty SLE patients in remission	Decreased Firmicutes/Bacteroidetes ratio. Reduction of Lachnospiraceae and Ruminococcaceae, enrichment of Bacteroidales.	Hevia et al., 2014

Psoriasis/ PsA	Fifteen patients with psoriasis of the skin; 16 PsA	Reduced diversity. *Coprococcus* decreased in psoriasis and PsA. *Akkermansia, Ruminococcus, Pseudobutyrivibrio* decreased in PsA.	Scher et al., 2015
	
	Twenty-nine psoriasis; 31 IBD; 13 concomitant psoriasis and IBD	Lower abundance of *F. prausnitzii* and higher abundance of *E. coli*	Eppinga et al., 2015

The largest study to date related to the mucosal-associated microbiota and new-onset CD conducted by Gevers et al. (2014) included 468 children and adolescents (<17 years) with newly diagnosed CD and a control group of 229 persons with non-inflammatory conditions of the gastrointestinal tract. Mucosal and fecal samples were obtained prior to the initiation of treatment in subjects with a spectrum of disease phenotypes based on severity, location (terminal ileum and rectum) and behavior. Overall, disease status strongly correlated to increases in the abundance of the Enterobacteriaceae, Pasteurellaceae, Veillonellaceae, and Fusobacteriaceae and a decrease in the abundance of Erysipelotrichales, Bacteroidales, and Clostridiales. Importantly, the alterations described were more strongly observed in tissue samples with weaker correlations observed in stool, implying a less dramatic shift in the luminal microbiota regardless of disease presence. The authors also demonstrated that microbiota profiles at time of diagnosis could be predictive for future disease outcomes; abundant levels of *Fusobacterium* and *Haemophilus* positively correlated with the pediatric CD activity index whereas levels of Enterobacteriaceae were negatively correlated. Furthermore, the microbiome composition between terminal ileal and rectal mucosal specimens were determined to be less different than differences observed between tissue and stool. Our group has recently reported similar observations of a somewhat homogenous gastrointestinal tract (using mucosal biopsies from the ileum, cecum, mid-colon, and rectum) in terms of microbe content and abundance within CD and UC cohorts (Forbes et al., 2016). We also explored the presence and absence of inflammation as an influencing factor on microbiome composition; shifts in microbe abundance comparing the inflamed mucosa between CD and UC were observed but these shifts were more dramatic in the non-inflamed mucosa between CD and UC.

### Microbes Enriched or Reduced in Inflammatory Bowel Disease

*Bacteroides* is the most dominant genus in Western microbiotas and can both positively and negatively affect the host (Wexler, 2007). Generally, while the overall abundance of the order Bacteroidales is increased in IBD, in certain circumstances particular species may be reduced; *Parabacteroides distasonis* is significantly decreased in inflamed IBD mucosa (Zitomersky et al., 2013). Pathogenic bacteria including *E. coli*, and *Shigella*, and others such as *Rhodococcus* and *Stenotrophomonas maltophilia* are increasingly observed in IBD (Knösel et al., 2009; Guerrero et al., 2015; Jensen et al., 2015). Other pathobionts with potential roles in the disease course include *Prevotellaceae, C. difficile, Klebsiella pneumoniae, Proteus mirabilis*, and *Helicobacter hepaticus*.

The relative abundance of the Enterobacteriaceae in persons with IBD (Kolho et al., 2015) and mouse models of IBD (Nagao-Kitamoto et al., 2016) is increased. Much research has focused on the role of *E. coli*, specifically AIEC, in IBD etiology; AIEC strains have been isolated from ileal-involving CD tissue (Darfeuille-Michaud et al., 2004) and the genera *Escherichia* and *Shigella* (indistinguishable by 16S analysis) are highly enriched in patients with IBD and this enrichment was more pronounced in mucosal samples versus stool samples (Petersen et al., 2015). It has also recently been demonstrated that a Western diet induces a shift in microbiome composition increasing the susceptibility to AIEC infection (Agus et al., 2016). AIEC possess the capability to invade enterocytes, replicate within macrophages and induce granuloma formation *in vitro* (Martinez-Medina and Garcia-Gil, 2014), and though AIEC strains may be present within a healthy gut, they do not have the ability to adhere to ileal enterocytes. Therefore, colonization of the inflamed ileum provides a preferential environment for these microbes to influence the disease course. It has also been shown that the anti-inflammatory drug, mesalamine, used for IBD therapy, decreases gut inflammation and correlates with a reduction of *Escherichia/Shigella* abundance (Morgan et al., 2012).

*Fusobacterium* species are members of the normal oral and gut microbiota in humans; however, particular species (adherent, invasive, pro-inflammatory) are recognized as opportunistic pathogens in CD and UC. The relative abundance of *Fusobacterium* is increased in mouse models and the colonic mucosa of IBD; Strauss et al. (2011) isolated *Fusobacterium* species from 63.6% of patients with IBD versus 26.5% of healthy controls. Moreover, 69% of all *Fusobacterium* isolated from IBD patients were identified as *F. nucleatum* and strains isolated from the IBD inflamed mucosa were more invasive in a Caco-2 cell invasion assay than strains isolated from the healthy mucosa of IBD or the control group (Strauss et al., 2011). *Fusobacterium* species have also been linked to colorectal carcinoma (Mima et al., 2015) and interestingly, IBD is a recognized risk factor for colorectal carcinoma (Lutgens et al., 2015).

The reduction of particular commensal microbes and a concomitant loss of their protective function possibly have a substantial impact on the course of disease. The ability of commensal bacteria to produce SCFA including butyrate, acetate and propionate via dietary fiber (a prebiotic) fermentation is a key benefit to the human gut. SCFA are a primary source of energy for colonic epithelial cells and have recently been shown to mediate homeostasis of colonic regulatory T cell populations (Smith et al., 2013). The abundance of *Faecalibacterium* of the Ruminococcaceae family is reduced in IBD (Lopez-Siles et al., 2015). *F. prausnitzii* is an anti-inflammatory commensal bacterium and produces the SCFA butyrate. *F. prausnitzii* administration in mouse models has been shown to reduce inflammation and secondly, a reduction in the abundance of *F. prausnitzii* has been linked to postoperative disease recurrence in ileal CD (Sokol et al., 2008). The abundance of other SCFA producers including the Leuconostocaceae, *Odoribacter splanchnius, Phascolarctobacterium*, and *Roseburia* are reduced in IBD (Morgan et al., 2012; Machiels et al., 2014). *Roseburia hominis*, in particular, inversely correlates to UC disease activity and duration (Machiels et al., 2014) and has been described as an acetate user and butyrate producer and is therefore dependent on other SCFA producers (Duncan et al., 2002). Alterations of these bacterial populations and concurrent variability of SCFA production may have profound consequences on host regulatory mechanisms of inflammation, though studies haven’t established causality.

### Multiple Sclerosis (MS)

Multiple sclerosis is a chronic inflammatory demyelinating disorder of the CNS. The cause of disease is not entirely understood, but both genetic and environmental components are involved in disease susceptibility (Dendrou et al., 2015). Though the CNS was originally regarded as an immune-privileged compartment, it is now recognized that immune cells survey the CNS regularly (Zhang et al., 1994); in MS and related neurological diseases there is an enrichment of autoreactive immune cells targeting elements of the CNS (myelin in MS). The discovery that gut microbes can produce neurotransmitters that can in turn influence the enteric nervous system and ultimately the CNS has led to the notion of the microbiota-gut-brain axis (Wang and Kasper, 2014).

Research investigating the role of the gut microbiome in experimental autoimmune encephalomyelitis (EAE), an animal model of MS, suggests a relationship between the gut microbes and development of MS. Ochoa-Repáraz et al. (2009) utilized a cocktail of oral antibiotics (ampicillin, vancomycin, neomycin sulfate, and metronidazole) to disrupt the gut microbiome of mice prior to induction of EAE. The authors reported oral antibiotic treatment significantly decreased gut bacterial populations and reduced the onset and severity of EAE. The reduced severity of EAE was ascribed to decreased levels of pro-inflammatory cytokines and chemokines and elevated levels of anti-inflammatory cytokines including IL-10 and IL-13; IL-10-producing Foxp3^+^ Treg cells were shown to accumulate in mesenteric and cervical lymph nodes and seemed to mediate the severity. Additionally, adoptive transfer of Foxp3^+^ Treg cells to naïve recipients conferred protection against disease. Furthermore, disease was observed among antibiotic treated mice with reduced CD25^+^ T cells. In contrast, in the absence of antibiotics, disease was exacerbated in CD25^+^-deficient mice. This implies regulatory populations other than Th1, Th17, or Treg cells may be pertinent to conferring protection against disease. An additional study in mice treated with oral antibiotics suggests a role for IL-10 producing CD19^+^ B cells (Ochoa-Repáraz et al., 2010a). Adoptive transfer from antibiotic treated mice to naïve recipient mice reduced severity of disease and this protective effect was attributed to shifting the immune response from a pro-inflammatory Th1/Th17 response to an anti-inflammatory Th2 response.

Lee et al. (2011) demonstrated disease protection in EAE is associated with reduced levels of the pro-inflammatory cytokines IFN-γ and IL-17A, both of which play critical roles in the development of EAE. Increased Foxp3^+^ Treg cells in the peripheral lymphoid tissues and the CNS were observed in germ-free mice. The authors further reported that SFB monocolonization of mice restored disease and examination of CNS tissues showed enhanced invasion by Th1 and Th17 cell populations. Using a relapsing-remitting mouse model (SJL/J) of spontaneously developing EAE, Berer et al. (2011) reported that the commensal gut microbiota is essential in triggering immunological processes that lead to relapsing-remitting autoimmune disease and that myelin-specific CD4^+^ T cells were driving factors of autoimmunity. Additionally, the activation of autoantibody-producing B cells was shown to be dependent upon the availability of the myelin autoantigen (specifically, MOG) and the gut microbiota.

The ability of probiotics to modify the clinical outcome of murine EAE has also been investigated. Prophylactic treatment with *Lactobacillus paracasei* and *L. plantarum* DSM 15312 was shown to induce Treg cells in mesenteric lymph nodes and enhance production of TGFβ1 whereas increased serum IL-27 levels were observed with *L. plantarum* DSM 15313 (Lavasani et al., 2010). Interestingly, administration of each strain independently was unsuccessful therapeutically whereas combinatorial administration suppressed disease perpetuation and reversed clinical signs of EAE. Disease amelioration was attributed to attenuation of the pro-inflammatory Th1/Th17 response and induction of Treg cells through elevated IL-10 levels. Comparably, an additional probiotic, *Bifidobacterium animalis* demonstrated similar suppressive effects (Ezendam et al., 2008). It has also been reported that some probiotic bacteria, notably *L. casei* Shirota, enhanced the duration of EAE (Ezendam and van Loveren, 2008); however, this was refuted in a subsequent study that determined neither *L. casei* Shirota nor *B. brevis* strain Yakult exacerbates EAE (Kobayashi et al., 2010). Prophylactic administration of five probiotics (IRT5; *L. casei, L. acidophilus, L. reuteni, B. bifidum*, and *Streptococcus thermophilus*) prior to disease induction significantly suppressed development of EAE (Kwon et al., 2013) whereas treatment with IRT5 to EAE delayed disease onset. Prophylactic and therapeutic efficacy of IRT5 probiotics was ascribed to inhibition of Th1 and Th17 responses and induction of IL-10-producing and/or Foxp3^+^ Treg cells.

To further explore how the microbiome affects EAE susceptibility and severity, the role of particular commensals or their products was considered. Ochoa-Repáraz et al. (2010b) investigated oral treatment of mice with polysaccharide A (PSA) of *B. fragilis.* In PSA treated mice, EAE severity was decreased relative to untreated mice and decreased numbers of Th1 and Th17 cells were reported in the EAE brains of PSA-treated mice. Moreover, lymphocytes isolated from the cervical lymph nodes of PSA-treated mice reportedly produced more IL-10 and less IL-17 and IFN-γ when stimulated with myelin oligodendrocyte glycoprotein (induction of EAE). Phosphorylated dihydroceramides, lipids derived from the oral commensal *P. gingivalis*, demonstrated enhanced EAE severity through induction of DC IL-6 secretion in a TLR2-dependent manner and a subsequent decrease in Foxp3^+^ Treg cell populations (Nichols et al., 2009). A recent study demonstrated oral administration of a *Lactococcus lactis* strain that produces heat shock protein-65 prevented development of murine EAE; the suppressive effect was ascribed to elevated IL-10 and reduced IL-17 production (Rezende et al., 2013).

Little is known about what impact the gut microbiome may have on human MS etiopathogenesis; gut microbiome studies are mostly limited to case-control examinations in persons with MS. Mowry et al. (2012) reported relapsing-remitting MS (RRMS) patients to have differing levels of Firmicutes, Bacteroidetes, and Proteobacteria, and that shifts in the abundance of Firmicutes was more dramatic in patients treated with glatiramer acetate, an immunomodulator drug. Comparatively, vitamin D treatment was associated with changes in Firmicutes, Actinobacteria, and Proteobacteria in RRMS patients, and increases in Enterobacteria in both RRMS and healthy controls. Vitamin D has been a main suspect in MS etiology for some time (Holmøy and Torkildsen, 2016). The beneficial aspects of vitamin D in the treatment of MS including exerting anti-inflammatory effects, possible influence on remyelination and its link to disease activity is widely accepted and is actively used as a therapeutic (Jarrett et al., 2014). The mechanism of benefit is unknown but it is plausible that it has an impact on the gut microbiome. Additional analysis by the same group revealed that although overlap of bacterial communities has been observed. Particular taxa including *Faecalibacterium* were reported to be lower in persons with MS (Cantarel et al., 2015). Of note, *Faecalibacterium* is considered to be immunosuppressive through production of the SCFA butyrate. Glatiramer acetate-treated patients showed differences in microbe composition including Bacteroidaceae, *Faecalibacterium, Ruminococcus*, Lactobacillaceae, *Clostridium*, and additional Clostridiales compared to untreated persons. After vitamin D supplementation, untreated persons with MS had an increase in *Akkermansia, Faecalibacterium*, and *Coprococcus.* Although these studies are exploratory and future studies in larger cohorts are needed to confirm these findings, these results suggest that the microbial communities of the MS gut are subject to modulation by some drugs commonly used in the treatment of MS. Jhangi et al. (2014) found a significant increase in the highly immunogenic Archaea *Methanobrevibacter smithii*, which might play a role in inflammation due to their structural components. *Butyricimonas* and Lachnospiraceae, which are butyrate producers with anti-inflammatory properties, were lower in untreated MS. Tremlett et al. (2015) explored gut microbiome profiles in pediatric MS; increases in *Escherichia, Shigella*, and *Clostridium* were observed, and decreases in *Eubacterium rectale* and *Corynebacterium.* Recently, the association between gut community profiles in 15 pediatric RRMS patients and host immunological markers such as IFN-γ, IL-17, IL-10, CD4^+^CD25^+^Foxp3^+^ Treg were explored (Tremlett et al., 2016a). The authors reported immune markers did not differ between RRMS and controls but did observe divergence in the association between the gut microbiome and immunological markers. IL-17^+^ T cells positively correlated with overall richness and evenness whereas an inverse correlation between IL-17^+^ T cells and Bacteroidetes abundance in MS was reported. Another study reporting on the association between the gut microbiota composition and relapse risk in pediatric MS showed a decrease in Fusobacteria, increase in Firmicutes and the presence of the Archaea Euryarchaeota was associated with a shorter time to relapse (Tremlett et al., 2016b). Thus far, current studies indicate that gut dysbiosis is linked with MS. Common findings include a reduction in the phyla Bacteroidetes and Firmicutes, which are important for immunoregulation and production of SCFA.

### Rheumatoid Arthritis (RA)

Rheumatoid arthritis is a common systemic autoimmune disease that manifests in the joints and if untreated can lead to chronic joint deformity, disability and increased mortality owing to cardiovascular and other concurrent, systemic complications. Notwithstanding recent advances in understanding the pathogenesis of RA (McInnes and Schett, 2011), the etiology remains undefined. Numerous genetic susceptibility risk alleles have been identified, but are insufficient to account for disease incidence. Therefore, RA is considered a multifactorial disease requiring both genetic and environmental factors for disease onset and/or progression.

Gut bacteria as an autoimmune trigger in RA has been recognized for decades. Mansson and Colldahl (1965) reported the majority of RA patients to have an intestinal flora with increased amounts of *Clostridium perfringens* type A in their feces. The patients also showed immune responses with elevated antibody titers to *C. perfringens* alphatoxin and positive skin tests. Ultimately, the elevation of *C. perfringens* was not specific to RA but was instead identified in a number of diseases, in particular, other chronic arthritides. It was later hypothesized that there are many bacteria involved in the pathogenesis of RA and not just one bacterium (Gullberg, 1978).

An accumulating body of literature exists to support a connection between gut bacteria and arthritis or more commonly referred to as the ‘gut–joint axis’ hypothesis. The groundbreaking study to identify the pro-arthritogenic role played by intestinal bacteria was reported by Kohashi et al. (1979) who discovered germ-free rats developed severe joint inflammation with 100% incidence in an adjuvant-induced arthritis model as compared to conventionally raised controls that developed less severe arthritis and at a modest incidence. This observation suggests that the gut microbiota may have important immunosuppressive effects. Other notable early findings supporting gut involvement in RA include studies reporting either protective or proarthritogenic roles of *E. coli* and *Bacteroides* species when introduced into germ-free arthritis-prone rats (Kohashi et al., 1985, 1986; Rath et al., 1996); germ-free *HLA-B27* transgenic rats remained unaffected from inflammatory (intestinal and peripheral) joint disease (Taurog et al., 1994), and germ-free rats become susceptible to arthritis –attributed to a loss of T-cell tolerance– in a streptococcal cell wall-induced rat arthritis model (van den Broek et al., 1992). More recently, Abdollahi-Roodsaz et al. (2008) determined arthritis is attenuated in IL-1 receptor antagonist-knockout (*Il1rn^-/-^*) mice (a model whereby mice spontaneously develop an autoimmune T-cell mediated arthritis). Applying gnotobiological methods, the introduction of the commensal species *Lactobacillus bifidus* resulted in disease onset. Development of arthritis in this model was attributed to an imbalance of Treg-Th17 homeostasis. Additional evidence comes from a study using the K/BxN T-cell receptor transgenic model of inflammatory arthritis (Wu et al., 2010). Germ-free mice did not develop disease, implying the gut microbiota is critical to the development of arthritis; protection of arthritis was ascribed to an absence of peripheral Th17 cells. Interestingly, monocolonization of germ-free K/Bxn mice with SFB effectively stimulated an autoimmune response thereby resulting in arthritis; autoimmunity developed as a result of promoting Th17 cells. Collectively, the above data derived from germ-free and gnotobiotic experimental animal models imply that the gut microbiota –particularly in a state of dysbiosis– is deemed necessary to trigger autoimmunity and consequent inflammatory arthritis. Moreover, the transition from a ‘normal’ gut microbiota to a state of dysbiosis likely requires a genetic predisposition.

One of the initial microbiome surveys in RA reported that persons early in disease course (disease duration ≤6 months) harbor significantly less bifidobacteria, *Bacteroides fragilis* subgroup, bacteria of the *Eubacterium rectale – Clostridium coccoides* subgroup and *Bacteroides–Porphyromonas–Prevotella* groups in comparison to persons with non-inflammatory fibromyalgia (Vaahtovuo et al., 2008). Another study specifically examined the relationship of *Lactobacillus* to RA (Liu et al., 2013); the authors identified significantly more *Lactobacillus* (10.62 ± 1.72 copies/g) in the RA gut compared to healthy controls (8.93 ± 1.60 copies/g). Furthermore, an increase in diversity and bacterial abundance was reported suggesting a possible relationship between *Lactobacillus* populations and disease onset and/or progression of RA. The abundance of *Lactobacillus* communities in RA, and other IMID, is relevant as these microorganisms and others are considered probiotic microbes that may confer a health benefit to the host. Sher et al. (2013) identified a strong association between *Prevotella copri* with new-onset untreated RA (NORA) and increases in the abundance of *Prevotella* corresponded with a reduction of several beneficial microbes, including the *Bacteroides*. Moreover, a negative correlation between the relative abundance of *P. copri* and the presence of shared epitope risk alleles was identified, indicating that the role of *P. copri* may play a more important role in persons with a lower load of genetic susceptibility. Zhang et al. (2015) recently analyzed the fecal, dental and salivary microbiome from treatment-naïve RA and unrelated healthy controls, as well as treatment-naïve RA paired with healthy relatives and DMARD-treated RA patients, representing the largest RA cohort and most comprehensive metagenomics analysis to date. The fecal analysis showed that *Haemophilus* spp. were depleted and *Lactobacillus salivarius* was over-represented in RA, and that more dramatic changes in abundance were observed in cases of active disease. In contrast, the abundance of *P. copri* was detected as a function of RA duration within the first year. Metagenomic linkage groups comprised of *Clostridium asparagiforme, Gordonibacter pamelaeae, Eggerthella lenta*, and *Lachnospiraceae bacterium* as well as *B. dentium, Lactobacillus* sp., and *Ruminococcus lactaris* were enriched in the RA gut. The investigators also showed that the altered gut microbiome could be used to identify RA patients, correlated with clinical indices [for example, titers of immunoglobulin A (IgA), major serum immunoglobulin (IgG), platelet count, anticyclic citrullinated peptide (anti-CCP) and rheumatoid factor (RF)], and could be utilized to stratify individuals based on their therapeutic response with disease-modifying anti-rheumatic drugs (DMARDs). And functionally, the redox environment and transport and metabolism of iron, zinc and sulfur were altered in persons with RA. A newly published study examined the microbial and metabolite profile in RA (Chen et al., 2016). RA patients were found to demonstrate a reduced microbial diversity and this correlated to disease duration and antibody levels. The gut in persons with RA was characterized by an expansion of the typically low-abundant phylum Actinobacteria and a decrease in more dominant phyla. The genera *Eggerthella* and *Actinomyces* of the Actinobacteria and *Turicibacter* and *Streptococcus* of the Firmicutes were significantly expanded in RA whereas the abundance of *Faecalibacterium* was significantly reduced. Moreover, the abundance of *Collinsella* correlated with high levels of alpha-aminoadipic acid and asparagine and IL-17A production. The study also confirmed the role of *Collinsella* in altering gut permeability and disease severity in experimental arthritis.

### Ankylosing Spondylitis (AS)

Ankylosing spondylitis belongs to a group of arthritides referred to as spondyloarthropathies. AS primarily targets the spine and sacroiliac joints and is characterized by enthesal inflammation. Disease progression is characterized by excessive bone formation (ankylosis) and eventually fuses joints, resulting in pain, stiffness, morbidity, and increased mortality (Thomas and Brown, 2010). The etiology of AS is unclear although environmental and genetic associations are considered to play a role. AS has been linked with the HLA-B27 allele since the 1970s (Schlosstein et al., 1973) and the recurrence risk among siblings and first degree relatives is 8.2% versus the general population with a risk of 0.1% (Brown et al., 2000).

Gut involvement in AS has been recognized for decades. Clinically, between 5 and 10% of persons with AS develop IBD at some time, and 70% of persons with AS are diagnosed with subclinical gut inflammation (Mielants et al., 1991). Relatively few studies have characterized the gut microbiome as it relates to AS. An early study by Stebbings et al. (2002) utilizing denaturing gradient gel electrophoresis reported no discernable differences in microbe populations of stool between 15 AS patients and 15 matched healthy controls as all subjects had unique and stable community profiles. Further analysis revealed a higher proportion of AS fecal samples contained sulfate-reducing bacteria [similar to IBD (Kushkevych, 2014)] compared to controls and a potential loss of immunological tolerance to autologous *Bacteroides*. Newly published evidence reveals a distinct microbial signature in the terminal ileum: Costello et al. (2015) recently surveyed the AS gut microbiome via 16S rRNA sequencing using terminal ileal biopsies from 9 AS patients and 9 controls. The terminal ileum of AS showed an enrichment of Lachnospiraceae, Ruminococcaceae, Rikenellaceae, Porphyromonadaceae, and Bacteroidaceae, a depletion of Veillonellaceae and Prevotellaceae and an overall increase in microbe diversity. Although the authors reported a decreased abundance of *Streptococcus* and *Actinomyces* in AS relative to healthy controls, they did not observe any differences in bacteria previously suspected to trigger AS, like *Klebsiella* species (Ebringer et al., 1977). Moreover, a recent study reported breast-feeding to induce protective effects on the development of AS (Montoya et al., 2016); interestingly, nutrition has a major impact on early microbiome composition and function (Backhed et al., 2015).

Animal models have demonstrated that gut dysbiosis plays a significant role in AS. Ankylosing enthesopathy (ANKENT) is a naturally occurring disease in mice with several parallels to human AS; in the ANKENT model, mice spontaneously develop ankylosis and enthesitis of the tarsal joint and ankle. Germ-free mice failed to develop ANKENT (Rehakova et al., 2000), however, mice colonized with common gut bacteria *Bacteroides, Enterococcus, Staphylococcus* and *Veillonella* developed joint disease but not when colonized with probacterium *Lactobacillus* (Sinkorova et al., 2008). Lin et al. (2014) characterized cecal microbiome differences mediated by the presence of the HLA-B27 gene (HLA-B27/human-ß2m transgenic rats versus wild-type Lewis rats) via biome representational *in situ* karyotyping (BRISK) and 16S rRNA sequencing. The BRISK analysis reported a higher prevalence of *Bacteroides vulgatus* in transgenic rats and 16S analysis showed increased abundance of *Paraprevotella* and decreased abundance of Rikenellaceae. The authors concluded that HLA-B27 is associated with a dysbiosis of the cecal microbiome. Another study by the same group reported an inflammatory cytokine signature and increased expression of antimicrobial peptides throughout the post-weaning period preceded the development of clinical bowel inflammation and dysbiosis in HLA-B27/ß2m rats (Asquith et al., 2016). Th17 populations were increased in cecal and colonic mucosa and also, increased *Akkermansia muciniphila* colonization and IgA coating of gut bacteria were correlated with HLA-B27 and clinical arthritis.

Another study explored the influence of bacterial cell wall components 1,3 ß-glucan (curdlan) or mannan on spondyloarthritis development. Using SKG mice (BALB/c ZAP-70^W163C^-mutants), Ruutu et al. (2012) reported ß-glucan to trigger spondyloarthritis and CD-like ileitis whereas mannan triggered spondylitis and arthritis which were T-cell and IL-23 dependent. The same group compared the responses of SKG mice (BALB/c ZAP-70^W163C^-mutants) deficient and wild-type for Toll-like receptor 4 to curdlan (Rehaume et al., 2014). Mice were raised under germ-free or specific pathogen free conditions and some mice were recolonized with altered Schaedler flora. The study demonstrated inflammation was present in all cases of curdlan exposure and that incidence and severity of arthritis/ileitis was dependent on microbiota content with severity of disease positively correlating with increased microbiome diversity.

### Systemic Lupus Erythematosus (SLE)

Systemic lupus erythematosus is a multi-system IMID with many clinical phenotypes and commonly involves the skin, joints, brain, kidneys, serosal surfaces, blood cells, blood vessels, heart, and lungs (Goldblatt and O’Neill, 2013). SLE affects persons with a wide spectrum of symptoms and disease courses including most frequently constitutional symptoms, malar (butterfly) rash, arthritis or arthralgia, renal and hematological symptoms (Goldblatt and O’Neill, 2013). From an immunological point of view, SLE demonstrates aberrant responses at varying levels: uncontrolled T-cell differentiation and activation, abnormal polyclonal B-cell activation and proliferation and autoantibody production linked to immune complex formation (Mok and Lau, 2003). It is the B-cell hyperactivity resulting in the production of several autoantibodies including those against double-stranded DNA, nucleosomes, phospholipids, *N*-methyl-D-aspartate receptor, C1q, and others (Goldblatt and O’Neill, 2013) that is a key pathological feature of SLE.

The role of the gut microbiome in SLE is an emerging field of research. Hevia et al. (2014) recently reported the first comprehensive characterization of the SLE gut microbiome: significant decrease in the Firmicutes/Bacteroidetes ratio was observed, similar to shifts observed in type 2 diabetes (Larsen et al., 2010) and CD (Manichanh et al., 2006). The change of Firmicutes/Bacteroidetes abundance in SLE was attributed to members of the orders Bacteroidales (Bacteroidetes) and Clostridiales (Firmicutes). Lachnospiraceae and Ruminococcaceae were positively associated with healthy controls. Some species of the Lachnospiraceae family like *Roseburia* sp. and *Butyrivibrio* sp. are butyrate producers; butyrate production in the human gut is highly relevant as it promotes Treg cell differentiation which can ultimately suppress pro-inflammatory responses (Singh et al., 2014). *Bacteroides* were significantly higher in SLE patients and *Desulfovibrio* were significantly reduced. The study also explored the functionality of the microbiome: glycan degradation pathways, lipopolysaccharide biosynthesis proteins and oxidative phosphorylation processes were associated with SLE partly explained by the increased abundance of Bacteroidetes, and more specifically *Bacteroides*. Another study by the same group performed a metabolome-wide scan of the gut microbiome (Rojo et al., 2015). The authors observed a slight difference (0.72% of metabolic mass features) in the gut metabolome but not in taxa composition and abundance of the gut microbiota. Cuervo et al. (2015) investigated the influence of dietary components to the gut microbiome of SLE. Positive correlations were identified between flavone and *Blautia*, flavanones, and *Lactobacillus*, and dihydrochalcones and *Bifidobacterium* in SLE; dihydroflavanols directly associated with *Faecalibacterium* and flavanol inversely correlated with *Bifidobacterium* in healthy controls.

Several studies utilizing murine models have demonstrated that gut dysbiosis might in fact be causal in the development of SLE. The effect of host genetics, sex, age, and dietary factors was recently investigated in a murine lupus model (Zhang et al., 2014). The female to male ratio for SLE is 10:1 and incidence rates at age 20–29 is highest (Jarukitsopa et al., 2015). Overall, young, female lupus-prone mice showed an increase in Lachnospiraceae and Bacteroidetes and a decrease in Bifidobacteriaceae and Erysipelotrichaceae. Diversity was also increased and lactobacilli were present in reduced numbers. The functional profile of lupus-prone mice revealed bacterial motility and sporulation-related pathways to be enriched. Retinoic acid as a dietary factor was found to restore lactobacilli populations, which correlated with reversing SLE functional pathways and improved symptoms. Additionally, the gut microbiome of lupus-prone mice differed between sexes; Lachnospiraceae were overrepresented in females that were found to correlate to earlier disease onset and/or increased diseases severity. A recent study assessing the impact of dietary factors on the development of SLE prone mice revealed significant differences in gut microbiome composition during pre-nephritic stages in mice drinking neutral (pH 7.0–7.2) versus acidic water (pH 3.0–3.2) and that mice drinking acidic water demonstrated lower levels of SLE-associated antibodies (anti-dsDNA and anti-nucleohistone), lower immune cell infiltrates in the kidney, and a slower progression of nephritis (Johnson et al., 2015).

### Psoriasis and Psoriatic Arthritis

Psoriasis is a chronic, inflammatory multi-organ disease, with a well-characterized and even pathognomonic pathology primarily manifesting in the skin and frequently the joints. The pathophysiology is dynamic and multifaceted, involving a complex interplay between genetics [HLA-Cw06 allele variant confers strongest susceptibility (Nair et al., 2006)], environmental triggers and both the innate and adaptive immune systems. Furthermore, approximately 24% of persons with psoriasis have concurrent psoriatic arthritis (Prey et al., 2010), a complex disorder involving peripheral and axial joints, dactylitis, enthesitis, and psoriasis.

Particular phenotypes of psoriasis have long been clinically linked to tonsillar infection with group A ß-haemolytic streptococci (Valdimarsson et al., 1995), and exacerbation of disease with skin or gut colonization by *Staphylococcus aureus* and fungi *Candida albicans* and *Malassezia* (Fry and Baker, 2007), however, studies investigating the correlation of psoriasis with the microbiome of the skin or gut are in their infancy. Fahlén et al. (2012) surveyed the cutaneous psoriasis microbiome via 16S rRNA sequencing. The study demonstrated that the Proteobacteria phylum was overrepresented and the abundance of Staphylococci and Propionibacteria was lower in psoriasis versus control skin. A recent study characterizing the gut microbiome reported an overall reduction of bacterial diversity and depletion of multiple (beneficial) gut bacteria in psoriasis and psoriatic arthritis and that changes in abundance were more dramatic in psoriatic arthritis (Scher et al., 2015). Both patient groups showed a decrease in *Coprococcus* species whereas psoriatic arthritis showed a reduction of *Akkermansia, Ruminococcus*, and *Pseudobutyrivibrio*. Interestingly, the authors determined that the gut microbiota profile of psoriatic arthritis is similar to the profile commonly observed in the IBD gut. Another study identified an IBD-like decrease of *Faecalibacterium prausnitzii* and increase of *E. coli* adding further support of a gut-skin axis (Eppinga et al., 2015). Gut dysbiosis in psoriatic arthritis has been shown to correlate to an increase of secretory IgA (sIgA) levels and a decrease of receptor activator of nuclear factor kappa-B ligand (RANK-L) levels within the gut luminal content (Scher et al., 2015). These results suggest that sIgA and/or RANK-L may be signs of a gut barrier breach due to dysbiosis or their possible role of disseminating inflammation to distant organs.

### The Gut Virome in Immune-Mediated Inflammatory Disease

To date, human microbiome studies have largely focused on bacterial components of the microbiome, though emerging data suggest that the viral components of the microbiome (virome) can have a profound impact on the host. Advances in sequencing technology and bioinformatics analysis have led to the discovery of a diverse human gut virome comprised of eukaryotic viruses and bacteriophages (viruses that infect bacteria) that outnumber human cells by 100-fold (Mokili et al., 2012). The functionality of the virome is not well defined although recent studies suggest the virome does in fact play an important role. It has been shown that a common enteric RNA virus, murine norovirus, can replace the beneficial function of commensal bacteria in germ-free or antibiotic-treated mice (Kernbauer et al., 2014). Importantly, eukaryotic viruses have demonstrated an interaction with IBD susceptibility genes to alter gut microbial populations as demonstrated in studies of mice carrying mutations in *Il-10* (Basic et al., 2014) or *Atg16l1* (Cadwell et al., 2010).

In the absence of disease, gut bacteriophage populations exhibit significant diversity and are predominated by members of the Caudovirales or Microviridae (Castro-Mejía et al., 2015). A number of studies have investigated the role of bacteriophages with respect to IBD. The abundance of bacteriophages in mucosal biopsies are significantly increased in CD versus healthy controls (Lepage et al., 2008), and are also more diverse in pediatric CD compared to healthy controls (Wagner et al., 2013). New evidence demonstrates that disease-specific alterations exist in the gut virome in IBD (Norman et al., 2015). This study showed that the IBD gut virome is abnormal; the gut virome richness is increased and a significant expansion of Caudovirales was observed in both CD and UC, but particular viromes of CD and UC were disease and cohort specific. Importantly, the expansion and diversification of the gut virome was found to be independent from alterations in bacterial populations.

As virome profiling in health and disease is a relatively new field, data are currently limited to investigations of IBD. With numerous viral infections as potential environmental triggers in IMID, particularly Epstein-Barr virus or cytomegalovirus in MS (Belbasis et al., 2015) and several other lines of evidence that indicates a role for viruses, we anticipate future studies will target the gut virome in other IMID.

### The Gut Mycobiome in Immune-Mediated Inflammatory Disease

The human fungal microbial populations (mycobiome) are now appreciated as complex and important for human health. Research of the mycobiome has only recently begun, particularly in health and in IBD. Fungal DNA accounts for approximately 0.2% of the mucosa-associated microbiota and 0.3% of the fecal microbiota (Ott et al., 2008). The fungal constituents of the healthy human gut have been characterized and were found to contain *Aspergillus, Candida, Cryptococcus, Penicillium, Pneumocystis*, and *Saccharomyces* (Dollive et al., 2012). In IBD, the fecal mycobiome was recently reported to be skewed (Sokol et al., 2016). Specifically, the authors observed an increased Basidiomycota/Ascomycota ratio, a reduced abundance of *Saccharomyces cerevisiae* and an increased abundance of *Candida albicans* relative to healthy controls. The study also explored the relationship between fungal and bacterial populations and suggests that CD may favor fungi at the expense of bacteria and that disease-specific inter-kingdom alterations exist. Mukhopadhya et al. (2014) studied the mycobiome in newly diagnosed pediatric IBD patients and found the phylum Basidiomycota to dominate in IBD whereas Ascomycota dominated in healthy controls. A higher fungal diversity has also been reported in CD but no disease-specific fungal species were reported in either CD or UC (Ott et al., 2008). Fungal abundance has also been linked to diet (Hoffmann et al., 2013); *Candida* positively associated with carbohydrate consumption and negatively associated with saturated fatty acid levels whereas *Aspergillus* negatively associated with SCFA levels in a carbohydrate-rich diet. Interestingly, anti-*Saccharomyces cerevisiae* antibodies (ASCA) have a role in diagnosis, disease phenotype and prognosis in CD (Russell et al., 2009).

Other domains of microorganisms including the archaea, fungi, or viruses are increasingly being recognized to have important functions in host health. However, in terms of studying the microbiota, research has been very bacteria-centric, as only a limited number of studies have investigated the micro-eukaryotes or viral components in IMID.

### Therapeutic Manipulation of the Gut Microbiome

The gut microbiota is increasingly being recognized as an attractive target for therapeutic intervention. Exposure to particular antibiotics is a known risk factor for development of some IMID, however, antibiotics may also impact on IMID disease course by reducing concentrations of luminal gut bacteria and subsequently alter the gut microbiota composition in a way that is beneficial to the host, resulting in the induction of remission and/or the prevention of relapse. For example, even in the absence of robust clinical evidence antibiotics have been considered by some as an effective therapy in IBD and routinely used as a therapeutic strategy. A diverse group of antibiotics including macrolides, fluoroquinolones, 5-nitroimidazoles, rifaximin, and antimycobacterial therapy either administered alone or in combination have been evaluated in clinical trials for IBD treatment. In a meta-analysis antibiotics were found to be superior to placebo (Khan et al., 2011). However, the antibiotics had such a disparate spectrum of activity the results of this meta analysis raises the question as to whether it is important to attack a specific spectrum of organisms or simply important to alter the gut microbiome in any way. Antibiotic treatment for RA has been utilized without a firm rationale as early as the 1930s with sulfasalazine, a combination of sulphapyridine and 5-aminosalicylic acid (Svartz, 1948), and later with tetracycline derivatives (Brown et al., 1971). A number of clinical trials have evaluated the use of minocycline (Kloppenburg et al., 1994; Tilley et al., 1995; O’Dell et al., 1997, 2001), macrolides (Saviola et al., 2002, 2013; Ogrendik, 2007a, 2009; Ogrendik and Karagoz, 2011) and levofloxacin (Ogrendik, 2007b) for the treatment of RA. Evidence concerning therapeutic use of antibiotics in MS is limited; use of minocycline for clinically isolated syndrome and RRMS is currently in phase III of clinical trials (Metz et al., 2009). There is currently no indication that antibiotics are beneficial in SLE or most spondyloarthropathies.

Repopulating the gut with a healthy community is an alternative approach to perturbing the existing microbiome, but so far, outcomes from these methods are controversial or not efficacious. Probiotics are dietary supplements that contain live microorganisms that when administered are thought to strengthen the existing gut microbiome. For IBD, the efficacy of probiotics in clinical outcomes demonstrates variable success. A recent meta-analysis of randomized controlled trials evaluating the effectiveness of probiotics reported probiotic supplementation is linked to inducing remission/response in active UC but not CD (Shen et al., 2014). And, only VSL#3 (a combination of *L. casei, L. plantarum, L. acidophilus, L. delbruekii* subsp. *bulgaricus, B. longim, B. breve, B. infantis*, and *S. salivarus* subsp. *thermophilus*) significantly increased remission/response rates. Studies indicate that probiotics cannot treat CD and UC with equal effectiveness suggesting altering a single parameter might not be enough to cure disease. There is clinical evidence that probiotics (*L. casei* 01) as adjunctive therapy improves disease activity and inflammatory status in patients with RA (Vaghef-Mehrabany et al., 2014). In contrast, a probiotic preparation containing *S. salivarius, B. lactis*, and *L. acidophilus* demonstrated no significant benefit in patients with spondyloarthritis (Jenks et al., 2010). There are currently no published clinical trials to date of probiotic administration in MS or SLE, though studies utilizing mice suggest a potential benefit in both SLE (Alard et al., 2009) and MS (discussed above).

Prebiotic refers to a food substance that may be fermented but not digested. The fermentation of prebiotics stimulates growth and activity of gut microbes potentially benefiting the host via production of energy and metabolic substrates. Studies evaluating the clinical use of prebiotics in specific diseases are limited, with the exception of CD. The administration of 15 g of fructo-oligosaccharides (inulin) in patients with active ileocolonic CD has shown both promising results (Lindsay et al., 2006) and no clinical benefit (Benjamin et al., 2011). A 10 g lactulose administered to active CD patients demonstrated no significant improvement (Hafer et al., 2007). Prebiotics may be bloating and may in themselves lead to gastrointestinal symptoms.

Fecal microbiota transplantation (FMT) is another approach that can be used in diseases linked to gut dysbiosis. FMT is a novel technique in which the gut microbiota are transferred from a healthy donor to the patient with the overall goal to introduce a stable microbial community in the gut. Until recently, FMT has primarily been utilized to successfully treat recurrent antibiotic-resistant *C. difficile* infection (van Nood et al., 2013). The clinical efficacy of FMT in UC is promising as FMT has been shown to induce remission in a greater percentage of patients than placebo and no difference in adverse events were reported (Moayyedi et al., 2015). FMT has been investigated in few other diseases including CD (Gordon and Harbord, 2014; Quera et al., 2014) as case reports, albeit data are too limited to determine clinical usefulness. More randomized controlled trials are warranted to evaluate donor selection and the frequency of FMT administration.

## Conclusion and Perspectives

Immune-mediated inflammatory diseases are multifaceted disorders that share many common underlying dynamics including epidemiological co-occurrence, genetic susceptibility, and numerous environmental factors. IMID are highly influenced by the structural and functional characteristics of the gut microbiome. Recent technological advancements have facilitated comprehensive analyses through large cohort studies and have expanded our knowledge of the structure and function of the gut in health and in many diseases. Most available gut microbiome research is correlative and experimental models for testing hypotheses emergent from correlative associations are necessary. Investigation of the microbiome and relevant mechanisms poses a significant challenge because of the complexity of the relationships between the microbiota with host genetics and environmental factors. Nevertheless, many microbiome surveys have focused on only a single IMID; future microbiome studies should include multiple disease cohorts to characterize the gut microbial populations simultaneously to accurately examine how particular microbial groups are altered between IMID.

## Author Contributions

JF participated in developing the idea for the article, drafting the article, and participated in the final editing. GV participated in developing the idea for the article and participated in the final editing. CB participated in developing the idea for the article and participated in the final editing.

## Conflict of Interest Statement

The authors declare that the research was conducted in the absence of any commercial or financial relationships that could be construed as a potential conflict of interest.

CB has served on advisory boards of AbbVie Canada, Janssen Canada, Pfizer Canada, Shire Canada, Takeda Canada, Cubist Pharmaceuticals, and has consulted to Mylan Pharmaceuticals. He has received unrestricted educational grants from AbbVie Canada, Janssen Canada, Shire Canada, and Takeda Canada. He is on speakers bureau of AbbVie Canada and Shire Canada.

## References

[B1] Abbott A. (2016). Scientists bust myth that our bodies have more bacteria than human cells. *Nature* 10.1038/nature.2016.19136

[B2] Abdollahi-Roodsaz S. Joosten L. A. B. Koenders M. I. Devesa I. Roelofs M. F. Radstake T. R. (2008). Stimulation of TLR2 and TLR4 differentially skews the balance of T cells in a mouse model of arthritis. *J. Clin. Invest.* 118 205–216. 10.1172/JCI32639 18060042 PMC2104479

[B3] Adams L. A. Morrison M. (2016). The microbiome in obesity, diabetes, and NAFLD: what is your gut telling us? *Curr. Hepatol. Rep.* 15 96–102. 10.1007/s11901-016-0299-5

[B4] Agus A. Denizot J. Thévenot J. Martinez-Medina M. Massier S. Sauvanet P. (2016). Western diet induces a shift in microbiota composition enhancing susceptibility to Adherent-Invasive *E. coli* infection and intestinal inflammation. *Sci. Rep.* 6:19032 10.1038/srep19032 PMC4705701 26742586

[B5] Alard P. Parnell S. Manirarora J. Kosiewicz M. Atay S. (2009). Probiotics control lupus progression via induction of regulatory cells and IL-10 production. *J. Immunol.* 182:30.

[B6] Ananthakrishnan A. N. (2015). Environmental risk factors for inflammatory bowel diseases: a review. *Dig. Dis. Sci.* 60 290–298. 10.1007/s10620-014-3350-9 25204669 PMC4304948

[B7] Arpaia N. Campbell C. Fan X. Dikiy S. van der Veeken J. deRoos P. (2013). Metabolites produced by commensal bacteria promote peripheral regulatory T-cell generation. *Nature* 504 451–455. 10.1038/nature12726 24226773 PMC3869884

[B8] Asquith M. Stauffer P. Davin S. Mitchell C. Lin P. Rosenbaum J. T. (2016). Perturbed mucosal immunity and dysbiosis accompany clinical disease in a rat model of spondyloarthritis. *Arthritis Rheumatol.* 10.1002/art.39681 [Epub ahead of print]. PMC5542398 26992013

[B9] Atarashi K. Tanoue T. Oshima K. Suda W. Nagano Y. Nishikawa H. (2013). Treg induction by a rationally selected mixture of Clostridia strains from the human microbiota. *Nature* 500 232–236. 10.1038/nature12331 23842501

[B10] Backhed F. Roswall J. Peng Y. Feng Q. Jia H. Kovatcheva-Datchary P. (2015). Dynamics and stabilization of the human gut microbiome during the first year of life. *Cell Host Microbe* 17 690–703. 10.1016/j.chom.2015.04.004 25974306

[B11] Basic M. Keubler L. M. Buettner M. Achard M. Breves G. Schröder B. (2014). Norovirus triggered microbiota-driven mucosal inflammation in interleukin 10-deficient mice. *Inflamm. Bowel Dis.* 20 431–443. 10.1097/01.MIB.0000441346.86827.ed 24487272

[B12] Bayry J. Radstake T. R. (2013). Immune-mediated inflammatory diseases: progress in molecular pathogenesis and therapeutic strategies. *Expert Rev. Clin. Immunol.* 9 297–299. 10.1586/eci.13.10 23557265

[B13] Becker C. Neurath M. F. Wirtz S. (2015). The intestinal microbiota in inflammatory bowel disease. *ILAR J.* 56 192–204. 10.1093/ilar/ilv030 26323629

[B14] Belbasis L. Bellou V. Evangelou E. Ioannidis J. P. A. Tzoulaki I. (2015). Environmental risk factors and multiple sclerosis: an umbrella review of systematic reviews and meta-analyses. *Lancet Neurol.* 14 263–273. 10.1016/S1474-4422(14)70267-4 25662901

[B15] Benjamin J. Hedin C. Koutsoumpa A. Ng S. McCarthy N. Hart A. (2011). Randomised, double-blind, placebo-controlled trial of fructo-oligosaccharides in active Crohn’s disease. *Gut* 60 923–929. 10.1136/gut.2010.232025 21262918

[B16] Berer K. Mues M. Koutrolos M. Rasbi Z. A. Boziki M. Johner C. (2011). Commensal microbiota and myelin autoantigen cooperate to trigger autoimmune demyelination - with comments. *Nature* 479 538–541. 10.1038/nature10554 22031325

[B17] Bernstein C. N. Blanchard J. Houston D. Wajda A. (2001a). The incidence of venous thromboembolic disease among patients with IBD: a population-based study. *Thromb. Haemost.* 85 430–434. 11307809

[B18] Bernstein C. N. Blanchard J. Rawsthorne P. Yu N. (2001b). The prevalence of extraintestinal diseases in inflammatory bowel disease: a population-based study. *Am. J. Gastroenterol.* 96 1116–1122. 10.1111/j.1572-0241.2001.03756.x 11316157

[B19] Bernstein C. N. Blanchard J. Wajda A. (2008). The incidence of arterial thromboembolic diseases in inflammatory bowel disease: a population-based study. *Clin. Gastroenterol. Hepatol.* 6 41–45. 10.1016/j.cgh.2007.09.016 18063423

[B20] Bernstein C. N. Blanchard J. F. Leslie W. Wajda A. Yu B. N. (2000). The incidence of fracture among patients with inflammatory bowel disease: a population-based cohort study. *Ann. Intern. Med.* 133 795–799. 10.7326/0003-4819-133-10-200011210-00012 11085842

[B21] Bernstein C. N. Wajda A. Blanchard J. F. (2005). The clustering of other chronic inflammatory diseases in inflammatory bowel disease: a population-based study. *Gastroenterology* 129 827–836. 10.1053/j.gastro.2005.06.021 16143122

[B22] Blasco-baque V. Garidou L. Pomié C. Escoula Q. Loubieres P. Le Gall-david S. (2016). Periodontitis induced by *Porphyromonas gingivalis* drives periodontal microbiota dysbiosis and insulin resistance via an impaired adaptive immune response. *Gut* 10.1136/gutjnl-2015-309897 [Epub ahead of print]. PMC5531227 26838600

[B23] Brown E. M. Wlodarska M. Willing B. P. Vonaesch P. Han J. Reynolds L. A. (2015). Diet and specific microbial exposure trigger features of environmental enteropathy in a novel murine model. *Nat. Commun.* 6:7806 10.1038/ncomms8806 PMC4532793 26241678

[B24] Brown M. A. Laval S. H. Brophy S. Calin A. (2000). Recurrence risk modelling of the genetic susceptibility to ankylosing spondylitis. *Ann. Rheum. Dis.* 59 883–886. 10.1136/ard.59.11.883 11053066 PMC1753017

[B25] Brown T. M. Clark H. W. Bailey J. S. Gray C. W. (1971). A mechanistic approach to treatment of rheumatoid type arthritis naturally occurring in a gorilla. *Trans. Am. Clin. Climatol. Assoc.* 82 227–247. 4104221 PMC2441056

[B26] Cadwell K. Patel K. K. Maloney N. S. Liu T. C. Ng A. C. Y. Storer C. E. (2010). Virus-plus-susceptibility gene interaction determines Crohn’s disease gene Atg16L1 phenotypes in intestine. *Cell* 141 1135–1145. 10.1016/j.cell.2010.05.009 20602997 PMC2908380

[B27] Cantarel B. L. Waubant E. Chehoud C. Kuczynski J. DeSantis T. Z. Warrington J. (2015). Gut microbiota in multiple sclerosis. *J. Investig. Med.* 63 729–734. 10.1097/JIM.0000000000000192 PMC4439263 25775034

[B28] Castro-Mejía J. L. Muhammed M. K. Kot W. Neve H. Franz C. M. Hansen L. H. (2015). Optimizing protocols for extraction of bacteriophages prior to metagenomic analyses of phage communities in the human gut. *Microbiome* 3:64 10.1186/s40168-015-0131-4 PMC4650499 26577924

[B29] Chen J. Wright K. Davis J. M. Jeraldo P. Marietta E. V. Murray J. (2016). An expansion of rare lineage intestinal microbes characterizes rheumatoid arthritis. *Genome Med.* 8:43 10.1186/s13073-016-0299-7 PMC4840970 27102666

[B30] Claesson M. J. Jeffery I. B. Conde S. Power S. E. O’Connor E. M. Cusack S. (2012). Gut microbiota composition correlates with diet and health in the elderly. *Nature* 488 178–184. 10.1038/nature11319 22797518

[B31] Cohen R. Robinson D. Paramore C. Fraeman K. Renahan K. Bala M. (2008). Autoimmune disease concomitance among inflammatory bowel disease patients in the United States, 2001-2002. *Inflamm. Bowel Dis.* 14 738–743. 10.1002/ibd.20406 18300281

[B32] Costello M. E. Ciccia F. Willner D. Warrington N. Robinson P. C. Gardiner B. (2015). Brief Report: intestinal dysbiosis in ankylosing spondylitis. *Arthritis Rheumatol.* 67 686–691. 10.1002/art.38967 25417597

[B33] Cucino C. Sonnenberg A. (2001). The comorbid occurrence of other diagnoses in patients with ulcerative colitis and Crohn’s disease. *Am. J. Gastroenterol.* 96 2107–2112. 10.1111/j.1572-0241.2001.03943.x 11467640

[B34] Cuervo A. Hevia A. López P. Suárez A. Sánchez B. Margolles A. (2015). Association of polyphenols from oranges and apples with specific intestinal microorganisms in systemic lupus erythematosus patients. *Nutrients* 7 1301–1317. 10.3390/nu7021301 25690419 PMC4344589

[B35] Darfeuille-Michaud A. Boudeau J. Bulois P. Neut C. Glasser A. L. Barnich N. (2004). High prevalence of adherent-invasive *Escherichia coli* associated with ileal mucosa in Crohn’s disease. *Gastroenterology* 127 412–421. 10.1053/j.gastro.2004.04.061 15300573

[B36] D’Argenio V. Casaburi G. Precone V. Pagliuca C. Colicchio R. Sarnataro D. (2016). Metagenomics reveals dysbiosis and a potentially pathogenic *N. flavescens* strain in duodenum of adult celiac patients. *Am. J. Gastroenterol.* 111 879–890. 10.1038/ajg.2016.95 27045926 PMC4897008

[B37] Daulatzai M. A. (2014). Obesity and gut’s dysbiosis promote neuroinflammation, cognitive impairment, and vulnerability to Alzheimer’ s disease: new directions and therapeutic implications. *Mol. Genet. Med.* S1:005 10.4172/1747-0862.S1-005

[B38] David L. A. Materna A. C. Friedman J. Campos-Baptista M. I. Blackburn M. C. Perrotta A. (2014). Host lifestyle affects human microbiota on daily timescales. *Genome Biol.* 15:R89 10.1186/gb-2014-15-7-r89 PMC4405912 25146375

[B39] de Meij T. de Groot E. Benninga M. Budding D. de Boer N. van Bodegraven A. (2015). Microbiota dynamics in paediatric Crohn’s disease from active disease upon achieving clinical remission. *J. Crohn’s Colitis* S437–S438. 10.1093/ecco-jcc/jju027.830

[B40] Dendrou C. A. Fugger L. Friese M. A. (2015). Immunopathology of multiple sclerosis. *Nat. Rev. Immunol.* 15 545–558. 10.1038/nri3871 26250739

[B41] Diaz-Gallo L.-M. Martin J. (2012). Common genes in autoimmune diseases: a link between immune-mediated diseases. *Expert Rev. Clin. Immunol.* 8 107–109. 10.1586/eci.11.90 22288446

[B42] Dicksved J. Halfvarson J. Rosenquist M. Jarnerot G. Tysk C. Apajalahti J. (2008). Molecular analysis of the gut microbiota of identical twins with Crohn’s disease. *ISME J.* 2 716–727. 10.1038/ismej.2008.37 18401439

[B43] Distrutti E. Monaldi L. Ricci P. Fiorucci S. Distrutti E. Monaldi L. (2016). Gut microbiota role in irritable bowel syndrome: new therapeutic strategies. *World J. Gastroenterol.* 22 2219–2241. 10.3748/wjg.v22.i7.2219 26900286 PMC4734998

[B44] Dollive S. Peterfreund G. L. Sherrill-Mix S. Bittinger K. Sinha R. Hoffmann C. (2012). A tool kit for quantifying eukaryotic rRNA gene sequences from human microbiome samples. *Genome Biol.* 13:R60 10.1186/gb-2012-13-7-r60 PMC4053730 22759449

[B45] Duncan S. H. Hold G. L. Barcenilla A. Stewart C. S. Flint H. J. (2002). *Roseburia intestinalis* sp. nov., a novel saccharolytic, butyrate-producing bacterium from human faeces. *Int. J. Syst. Evol. Microbiol.* 52 1615–1620. 10.1099/ijs.0.02143-0 12361264

[B46] Ebringer R. Cooke D. Cawdell D. R. Cowling P. Ebringer A. (1977). Ankylosing spondylitis: *Klebsiella* and HL-A B27. *Rheumatol. Rehabil.* 16 190–196. 10.1093/rheumatology/16.3.190 910095

[B47] Eppinga H. Weiland C. J. S. Bing Thio H. van der Woude C. J. Nijsten T. E. C. Peppelenbosch M. P. (2015). Similar depletion of protective *Faecalibacterium prausnitzii* in psoriasis and inflammatory bowel disease, but not in Hidradenitis suppurativa. *J. Crohn’s Colitis* 10.1093/ecco-jcc/jjw070 [Epub ahead of print]. 26971052

[B48] Ezendam J. De Klerk A. Gremmer E. R. Van Loveren H. (2008). Effects of Bifidobacterium animalis administered during lactation on allergic and autoimmune responses in rodents. *Clin. Exp. Immunol.* 154 424–431. 10.1111/j.1365-2249.2008.03788.x 19037925 PMC2633237

[B49] Ezendam J. van Loveren H. (2008). *Lactobacillus casei* Shirota administered during lactation increases the duration of autoimmunity in rats and enhances lung inflammation in mice. *Br. J. Nutr.* 99 83–90. 10.1017/S0007114507803412 17678568

[B50] Fahlén A. Engstrand L. Baker B. S. Powles A. Fry L. (2012). Comparison of bacterial microbiota in skin biopsies from normal and psoriatic skin. *Arch. Dermatol. Res.* 304 15–22. 10.1007/s00403-011-1189-x 22065152

[B51] Faith J. J. Guruge J. L. Charbonneau M. Subramanian S. Seedorf H. Goodman A. L. (2013). The long-term stability of the human gut microbiota. *Science* 341:1237439 10.1126/science.1237439 PMC3791589 23828941

[B52] Forbes J. D. Van Domselaar G. Bernstein C. N. (2016). Microbiome survey of the inflamed and noninflamed gut at different compartments within the gastrointestinal tract of inflammatory bowel disease patients. *Inflamm. Bowel Dis.* 22 817–825. 10.1097/MIB.0000000000000684 26937623

[B53] Fry L. Baker B. S. (2007). Triggering psoriasis: the role of infections and medications. *Clin. Dermatol.* 25 606–615. 10.1016/j.clindermatol.2007.08.015 18021899

[B54] Gaboriau-Routhiau V. Rakotobe S. Lécuyer E. Mulder I. Lan A. Bridonneau C. (2009). The key role of segmented filamentous bacteria in the coordinated maturation of gut helper T cell responses. *Immunity* 31 677–689. 10.1016/j.immuni.2009.08.020 19833089

[B55] Gevers D. Kugathasan S. Denson L. A. Vázquez-Baeza Y. Van Treuren W. Ren B. (2014). The treatment-naive microbiome in new-onset Crohn’s disease. *Cell Host Microbe* 15 382–392. 10.1016/j.chom.2014.02.005 24629344 PMC4059512

[B56] Goldblatt F. O’Neill S. G. (2013). Clinical aspects of autoimmune rheumatic diseases. *Lancet* 382 797–808. 10.1016/S0140-6736(13)61499-3 23993190

[B57] Gordon H. Harbord M. (2014). A patient with severe Crohn’s colitis responds to faecal microbiota transplantation. *J. Crohn’s Colitis* 8 256–257. 10.1016/j.crohns.2013.10.007 24239403

[B58] Guerrero C. Tort J. Pérez J. Andrés M. Espejo E. (2015). Rhodococcus equi infection in a patient with Crohn’s disease treated with infliximab. *J. Infect.* 70 689–690. 10.1016/j.jinf.2014.12.008 25546345

[B59] Gullberg R. (1978). Possible role of alterations of the intestinal flora in Rheumatoid Arthritis. *Rheumatol. Rehabil.* XVII, Suppl 5–10. 10.1093/rheumatology/XVII.suppl.5 364611

[B60] Hafer A. Krämer S. Duncker S. Krüger M. Manns M. P. Bischoff S. C. (2007). Effect of oral lactulose on clinical and immunohistochemical parameters in patients with inflammatory bowel disease: a pilot study. *BMC Gastroenterol.* 7:36 10.1186/1471-230X-7-36 PMC1995200 17784949

[B61] Harbord M. Annese V. Vavricka S. R. Allez M. Barreiro-De Acosta M. Boberg K. M. (2016). The First European evidence-based consensus on extra-intestinal manifestations in inflammatory bowel disease. *J. Crohn’s Colitis* 10 239–254. 10.1093/ecco-jcc/jjv213 26614685 PMC4957476

[B62] Hevia A. Milani C. López P. Cuervo A. Arboleya S. Duranti S. (2014). Intestinal dysbiosis associated with systemic lupus erythematosus. *MBio* 5:e1548-14 10.1128/mBio.01548-14 PMC4196225 25271284

[B63] Hoffmann C. Dollive S. Grunberg S. Chen J. Li H. Wu G. D. (2013). Archaea and fungi of the human gut microbiome: correlations with diet and bacterial residents. *PLoS ONE* 8:e66019 10.1371/journal.pone.0066019 PMC3684604 23799070

[B64] Holmøy T. Torkildsen Ø. (2016). Can vitamin D reduce inflammation in relapsing-remitting multiple sclerosis? *Expert Rev. Neurother.* 16 233–235. 10.1586/14737175.2016.1146134 26796244

[B65] Huttenhower C. Gevers D. Knight R. Abubucker S. Badger J. H. Chinwalla A. T. (2012). Structure, function and diversity of the healthy human microbiome. *Nature* 486 207–214. 10.1038/nature11234 22699609 PMC3564958

[B66] Jarrett F. Ducasa G. M. Buller D. B. Berwick M. (2014). The effect of oral supplementation of vitamin D3 on serum levels of vitamin D: a review. *Epidemiol. Open Access* 04 2–6. 10.4172/2161-1165.1000148

[B67] Jarukitsopa S. Hoganson D. D. Crowson C. S. Sokumbi O. Davis M. D. Michet C. J. Jr. (2015). Epidemiology of systemic lupus erythematosus and cutaneous lupus erythematosus in a predominantly white population in the United States. *Arthritis Care Res.* 67 817–828. 10.1002/acr.22502 PMC4418944 25369985

[B68] Jenks K. Stebbings S. Burton J. Schultz M. Herbison P. Highton J. (2010). Probiotic therapy for the treatment of spondyloarthritis: a randomized controlled trial. *J. Rheumatol.* 37 2118–2125. 10.3899/jrheum.100193 20716665

[B69] Jensen S. R. Mirsepasi-Lauridsen H. C. Thysen A. H. Brynskov J. Krogfelt K. A. Petersen A. M. (2015). Distinct inflammatory and cytopathic characteristics of *Escherichia coli* isolates from inflammatory bowel disease patients. *Int. J. Med. Microbiol.* 305 925–936. 10.1016/j.ijmm.2015.10.002 26522075

[B70] Jhangi S. Gandhi R. Glanz B. Cook S. Nejad P. Ward D. (2014). Increased Archaea species and changes with therapy in gut microbiome of multiple sclerosis subjects (S24. 001). *Neurology* 82:S24.001.

[B71] Johnson B. M. Gaudreau M. C. Al-Gadban M. M. Gudi R. Vasu C. (2015). Impact of dietary deviation on disease progression and gut microbiome composition in lupus-prone SNF1 mice. *Clin. Exp. Immunol.* 181 323–337. 10.1111/cei.12609 25703185 PMC4516448

[B72] Johnson C. C. Ownby D. R. (2016). Allergies and asthma: do atopic disorders result from inadequate immune homeostasis arising from infant gut dysbiosis? *Expert Rev. Clin. Immunol.* 12 379–388. 10.1586/1744666X.2016.1139452 26776722 PMC4829075

[B73] Kang S. Denman S. E. Morrison M. Yu Z. Dore J. Leclerc M. (2010). Dysbiosis of fecal microbiota in Crohn’s disease patients as revealed by a custom phylogenetic microarray. *Inflamm. Bowel Dis.* 16 2034–2042. 10.1002/ibd.21319 20848492

[B74] Keely S. Walker M. M. Marks E. Talley N. J. (2015). Immune dysregulation in the functional GI disorders. *Eur. J. Clin. Invest.* 45 1350–1359. 10.1111/eci.12548 26444549

[B75] Kernbauer E. Ding Y. Cadwell K. (2014). An enteric virus can replace the beneficial function of commensal bacteria. *Nature* 516 94–98. 10.1038/nature13960 25409145 PMC4257755

[B76] Khan K. J. Ullman T. A. Ford A. C. Abreu M. T. Abadir A. Marshall J. K. (2011). Antibiotic therapy in inflammatory bowel disease: a systematic review and meta-analysis. *Am. J. Gastroenterol.* 106 661–673. 10.1038/ajg.2011.72 21407187

[B77] Kloppenburg M. Breedveld F. C. Terwiel J. P. Mallee C. Dijkmans B. A. (1994). Minocycline in active rheumatoid arthritis. *Arthritis Rheumatol.* 37 629–636. 10.1002/art.1780370505 8185689

[B78] Knösel T. Schewe C. Petersen N. Dietel M. Petersen I. (2009). Prevalence of infectious pathogens in Crohn’s disease. *Pathol. Res. Pract.* 205 223–230. 10.1016/j.prp.2008.04.018 19186006

[B79] Kobayashi T. Kato I. Nanno M. Shida K. Shibuya K. Matsuoka Y. (2010). Oral administration of probiotic bacteria, *Lactobacillus casei* and *Bifidobacterium breve*, does not exacerbate neurological symptoms in experimental autoimmune encephalomyelitis. *Immunopharmacol. Immunotoxicol.* 32 116–124. 10.3109/08923970903200716 19831500

[B80] Kohashi O. Kohashi Y. Takahashi H. Ozawa A. Shigematsu N. (1985). Reverse effect of gram-positive bacteria vs. gram-negative bacteria on adjuvant-induced arthritis in germfree rats. *Microbiol. Immunol.* 29 487–497. 10.1111/j.1348-0421.1985.tb00851.x 2931580

[B81] Kohashi O. Kohashi Y. Takahashi H. Ozawa A. Shigematsu N. (1986). Suppressive effect of *Escherichia coli* on adjuvant-induced arthritis in germ-free ratse. *Arthritis Rheum.* 29 547–553. 10.1002/art.1780290413 3518723

[B82] Kohashi O. Kuwata J. Umehara K. Uemura F. Takahashi T. Ozawa A. (1979). Susceptibility to adjuvant-induced arthritis among germfree, specific-pathogen-free, and conventional rats. *Infect. Immun.* 26 791–794. 160888 10.1128/iai.26.3.791-794.1979 PMC414687

[B83] Kolho K.-L. Korpela K. Jaakkola T. Pichai M. V. A. Zoetendal E. G. Salonen A. (2015). Fecal microbiota in pediatric inflammatory bowel disease and its relation to inflammation. *Am. J. Gastroenterol.* 110 921–930. 10.1038/ajg.2015.149 25986361

[B84] Kushkevych V. I (2014). Etiological role of sulfate-reducing bacteria in the development of inflammatory bowel diseases and Ulcerative Colitis. *Am. J. Infect. Dis. Microbiol.* 2 63–73. 10.12691/ajidm-2-3-5

[B85] Kwon H. K. Kim G. C. Kim Y. Hwang W. Jash A. Sahoo A. (2013). Amelioration of experimental autoimmune encephalomyelitis by probiotic mixture is mediated by a shift in T helper cell immune response. *Clin. Immunol.* 146 217–227. 10.1016/j.clim.2013.01.001 23416238

[B86] Larcombe S. Hutton M. L. Lyras D. (2016). Involvement of bacteria other than *clostridium* difficile in antibiotic-associated diarrhoea. *Trends Microbiol.* 24 463–476. 10.1016/j.tim.2016.02.001 26897710

[B87] Larsen N. Vogensen F. K. Van Den Berg F. W. J. Nielsen D. S. Andreasen A. S. Pedersen B. K. (2010). Gut microbiota in human adults with type 2 diabetes differs from non-diabetic adults. *PLoS ONE* 5:e9085 10.1371/journal.pone.0009085 PMC2816710 20140211

[B88] Lavasani S. Dzhambazov B. Nouri M. Fåk F. Buske S. Molin G. (2010). A novel probiotic mixture exerts a therapeutic effect on experimental autoimmune encephalomyelitis mediated by IL-10 producing regulatory T cells. *PLoS ONE* 5:e9009 10.1371/journal.pone.0009009 PMC2814855 20126401

[B89] Lee Y. K. Menezes J. S. Umesaki Y. Mazmanian S. K. (2011). Proinflammatory T-cell responses to gut microbiota promote experimental autoimmune encephalomyelitis. *Proc. Natl. Acad. Sci. U.S.A.* 108(Suppl.) 4615–4622. 10.1073/pnas.1000082107 20660719 PMC3063590

[B90] Lepage P. Colombet J. Marteau P. Sime-Ngando T. Dore J. Leclerc M. (2008). Dysbiosis in inflammatory bowel disease: a role for bacteriophages? *Gut* 57 424–425. 10.1136/gut.2007.134668 18268057

[B91] Lepage P. Hösler R. Spehlmann M. E. Rehman A. Zvirbliene A. Begun A. (2011). Twin study indicates loss of interaction between microbiota and mucosa of patients with ulcerative colitis. *Gastroenterology* 141 227–236. 10.1053/j.gastro.2011.04.011 21621540

[B92] Li G. Yang M. Zhou K. Zhang L. Tian L. Lv S. (2015). Diversity of duodenal and rectal microbiota in biopsy tissues and luminal contents in healthy volunteers. *J. Microbiol. Biotechnol.* 25 1136–1145. 10.4014/jmb.1412.12047 25737115

[B93] Lin P. Bach M. Asquith M. Lee A. Y. Akileswaran L. Stauffer P. (2014). HLA-B27 and human β2-microglobulin affect the gut microbiota of transgenic rats. *PLoS ONE* 9:e105684 10.1371/journal.pone.0105684 PMC4139385 25140823

[B94] Lindsay J. O. Whelan K. Stagg A. J. Gobin P. Al-Hassi H. O. Rayment N. (2006). Clinical, microbiological, and immunological effects of fructo-oligosaccharide in patients with Crohn’s disease. *Gut* 55 348–355. 10.1136/gut.2005.074971 16162680 PMC1856087

[B95] Liu X. Zou Q. Zeng B. Fang Y. Wei H. (2013). Analysis of fecal Lactobacillus community structure in patients with early rheumatoid arthritis. *Curr. Microbiol.* 67 170–176. 10.1007/s00284-013-0338-1 23483307

[B96] Lopez-Siles M. Martinez-Medina M. Abella C. Busquets D. Sabat-Mir M. Duncan S. H. (2015). Mucosa-associated *Faecalibacterium prausnitzii* phylotype richness is reduced in patients with inflammatory bowel disease. *Appl. Environ. Microbiol.* 81 7582–7592. 10.1128/AEM.02006-15 26296733 PMC4592880

[B97] Lutgens M. Vermeire S. Van Oijen M. Vleggaar F. Siersema P. van Assche G. (2015). A rule for determining risk of colorectal cancer in patients with inflammatory bowel disease. *Clin. Gastroenterol. Hepatol.* 13 148.e1–154.e1. 10.1016/j.cgh.2014.06.032 25041864

[B98] Machiels K. Joossens M. Sabino J. De Preter V. Arijs I. Eeckhaut V. (2014). A decrease of the butyrate-producing species *Roseburia hominis* and *Faecalibacterium prausnitzii* defines dysbiosis in patients with ulcerative colitis. *Gut* 63 1275–1283. 10.1136/gutjnl-2013-304833 24021287

[B99] Manichanh C. Rigottier-Gois L. Bonnaud E. Gloux K. Pelletier E. Frangeul L. (2006). Reduced diversity of faecal microbiota in Crohn’s disease revealed by a metagenomic approach. *Gut* 55 205–211. 10.1136/gut.2005.073817 16188921 PMC1856500

[B100] Mansson I. Colldahl H. (1965). The intestinal flora in patients with bronchial asthma and rheumatoid arthritis. *Allergy* 20 94–104. 10.1111/j.1398-9995.1965.tb03360.x 14311534

[B101] Marin-Jiminez I. García Sánchez V. Gisbert J. Lázaro Pérez Calle J. Luján M. Gordillo Ábalos J. (2014). Prevalence of different immune mediated inflammatory diseases in patients with inflammatory bowel disease. AQUILES study. *Gastroenterol. Hepatol.* 37 495–502. 10.1016/S1873-9946(13)60647-9 24717523

[B102] Martinez-Medina M. Garcia-Gil L. (2014). *Escherichia coli* in chronic inflammatory bowel diseases: an update on adherent invasive *Escherichia coli* pathogenicity. *World J. Gastrointest. Pathophysiol.* 5 213–227. 10.4291/wjgp.v5.i3.213 25133024 PMC4133521

[B103] McInnes I. B. Schett G. (2011). The pathogenesis of rheumatoid arthritis. *N. Engl. J. Med.* 365 2205–2219. 10.1056/NEJMra1004965 22150039

[B104] Metz L. M. Li D. Traboulsee A. Myles M. L. Duquette P. Godin J. (2009). Glatiramer acetate in combination with minocycline in patients with relapsing–remitting multiple sclerosis: results of a Canadian, multicenter, double-blind, placebo-controlled trial. *Mult. Scler.* 15 1183–1194. 10.1177/1352458509106779 19776092

[B105] Mielants H. Veys E. Goemaere S. Goethals K. Cuvelier C. De Vos M. (1991). Gut inflammation in the spondyloarthropathies: clinical, radiologic, biologic and genetic features in relation to the type of histology. A prospective study. *J. Rheumatol.* 18 1542–1551. 1765980

[B106] Mima K. Nishihara R. Qian Z. R. Cao Y. Sukawa Y. Nowak J. A. (2015). *Fusobacterium nucleatum* in colorectal carcinoma tissue and patient prognosis. *Gut* 10.1136/gutjnl-2015-310101 [Epub ahead of print]. PMC4769120 26311717

[B107] Moayyedi P. Surette M. G. Kim P. T. Libertucci J. Wolfe M. Onischi C. (2015). Fecal microbiota transplantation induces remission in patients with active ulcerative colitis in a randomized controlled trial. *Gastroenterology* 149 102–109. 10.1053/j.gastro.2015.04.001 25857665

[B108] Mok C. Lau C. (2003). Pathogenesis of systemic lupus erythematosus. *J. Clin. Pathol.* 56 481–490. 10.1136/jcp.56.7.481 12835292 PMC1769989

[B109] Mokili J. L. Rohwer F. Dutilh B. E. (2012). Metagenomics and future perspectives in virus discovery. *Curr. Opin. Virol.* 2 63–77. 10.1016/j.coviro.2011.12.004 22440968 PMC7102772

[B110] Mondot S. De Wouters T. Doré J. Lepage P. (2013). The human gut microbiome and its dysfunctions. *Dig. Dis.* 31 278–285. 10.1159/000354678 24246975

[B111] Montoya J. Matta N. B. Suchon P. Guzian M. C. Lambert N. C. Mattei J. P. (2016). Patients with ankylosing spondylitis have been breast fed less often than healthy controls: a case–control retrospective study. *Ann. Rheum. Dis.* 75 879–882. 10.1136/annrheumdis-2015-208187 26458738

[B112] Morgan X. C. Tickle T. L. Sokol H. Gevers D. Devaney K. L. Ward D. V. (2012). Dysfunction of the intestinal microbiome in inflammatory bowel disease and treatment. *Genome Biol.* 13:R79 10.1186/gb-2012-13-9-r79 PMC3506950 23013615

[B113] Mowry E. Waubant E. Chehoud C. DeSantis T. Kuczynski J. Warrington J. (2012). Gut bacterial populations in multiple sclerosis and in health (P05.106). *Neurology* 78:P05.106 10.1212/WNL.78.1_MeetingAbstracts.P05.106

[B114] Mucida D. Park Y. Kim G. Turovskaya O. Scott I. Kronenberg M. (2007). Reciprocal Th17 and regulatory T cell differentiation mediated by retinoic acid. *Science* 317 256–260. 10.1126/science.1140114 17569825

[B115] Mukhopadhya I. Hansen R. Meharg C. Thomson J. M. Russell R. K. Berry S. H. (2014). The fungal microbiota of de-novo paediatric inflammatory bowel disease. *Microbes Infect.* 17 304–310. 10.1016/j.micinf.2014.12.001 25522934 PMC4392392

[B116] Naftali T. Reshef L. Kovacs A. Porat R. Amir I. Konikoff F. M. (2016). Distinct microbiotas are associated with ileum-restricted and colon-involving Crohn’s disease. *Inflamm. Bowel Dis.* 22 293–302. 10.1097/MIB.0000000000000662 26752462

[B117] Nagao-Kitamoto H. Shreiner A. B. Gillilland M. G. Kitamoto S. Ishii C. Hirayama A. (2016). Functional characterization of inflammatory bowel disease–associated gut dysbiosis in gnotobiotic mice. *CMGH Cell. Mol. Gastroenterol. Hepatol.* 2 468–481. 10.1016/j.jcmgh.2016.02.003 PMC5042563 27795980

[B118] Nair R. P. Stuart P. E. Nistor I. Hiremagalore R. Chia N. V. C. Jenisch S. (2006). Sequence and haplotype analysis supports HLA-C as the psoriasis susceptibility 1 gene. *Am. J. Hum. Genet.* 7878 827–851. 10.1086/503821 16642438 PMC1474031

[B119] Nichols F. C. Housley W. J. O’Conor C. A. Manning T. Wu S. Clark R. B. (2009). Unique lipids from a common human bacterium represent a new class of Toll-like receptor 2 ligands capable of enhancing autoimmunity. *Am. J. Pathol.* 175 2430–2438. 10.2353/ajpath.2009.090544 19850890 PMC2789629

[B120] Norman J. M. Handley S. A. Parkes M. Virgin H. W. Norman J. M. Handley S. A. (2015). Disease-specific alterations in the enteric virome in article disease-specific alterations in the enteric virome in inflammatory bowel disease. *Cell* 160 447–460. 10.1016/j.cell.2015.01.002 25619688 PMC4312520

[B121] Ochoa-Repáraz J. Mielcarz D. W. Ditrio L. E. Burroughs A. R. Foureau D. M. Haque-Begum S. (2009). Role of gut commensal microflora in the development of experimental autoimmune encephalomyelitis. *J. Immunol.* 183 6041–6050. 10.4049/jimmunol.0900747 19841183

[B122] Ochoa-Repáraz J. Mielcarz D. W. Haque-Begum S. Kasper L. H. (2010a). Induction of a regulatory B cell population in experimental allergic encephalomyelitis by alteration of the gut commensal microflora. *Gut Microbes* 1 103–108. 10.4161/gmic.1.2.11515 21326918 PMC3023588

[B123] Ochoa-Repáraz J. Mielcarz D. W. Wang Y. Begum-Haque S. Dasgupta S. Kasper D. L. (2010b). A polysaccharide from the human commensal *Bacteroides fragilis* protects against CNS demyelinating disease. *Mucosal Immunol.* 3 487–495. 10.1038/mi.2010.29 20531465

[B124] O’Dell J. R. Blakely K. W. Mallek J. A. Eckhoff P. J. Leff R. D. Wees S. J. (2001). Treatment of early seropositive rheumatoid arthritis: a two year, double blind comparison of minocycline and hydroxychloroquine. *Arthritis Rheum.* 44 2235–2241. 10.1002/1529-0131(200110)44:10<2235::AID-ART385>3.0.CO;2-A 11665963

[B125] O’Dell J. R. Haire C. E. Palmer W. Drymalski W. Wees S. Blakely K. (1997). Treatment of early rheumatoid arthritis with minocycline or placebo: results of a randomized, double-blind, placebo-controlled trial. *Arthritis Rheum.* 40 842–848. 10.1002/art.1780400510 9153544

[B126] Ogrendik M. (2007a). Effects of clarithromycin in patients with active rheumatoid arthritis. *Curr. Med. Res. Opin.* 23 515–522. 10.1185/030079906X167642 17355733

[B127] Ogrendik M. (2007b). Levofloxacin treatment in patients with rheumatoid arthritis receiving methotrexate. *South Med. J.* 100 135–139. 10.1097/01.smj.0000254190.54327.3b 17330681

[B128] Ogrendik M. (2009). Efficacy of roxithromycin in adult patients with rheumatoid arthritis who had not received disease-modifying antirheumatic drugs: a 3-month, randomized, double-blind, placebo-controlled trial. *Clin. Ther.* 31 1754–1764. 10.1016/j.clinthera.2009.08.014 19808134

[B129] Ogrendik M. Karagoz N. (2011). Treatment of rheumatoid arthritis with roxithromycin: a randomized trial. *Postgrad. Med.* 123 220–227. 10.3810/pgm.2011.09.2478 21904105

[B130] Ott S. J. Kuhbacher T. Musfeldt M. Rosenstiel P. Hellmig S. Rehman A. (2008). Fungi and inflammatory bowel diseases: alterations of composition and diversity. *Scand. J. Gastroenterol.* 43 831–841. 10.1080/00365520801935434 18584522

[B131] Parkes M. Cortes A. van Heel D. A. Brown M. A. (2013). Genetic insights into common pathways and complex relationships among immune-mediated diseases. *Nat. Rev. Genet.* 14 661–673. 10.1038/nrg3502 23917628

[B132] Petersen A. M. Halkjær S. I. Gluud L. L. (2015). Intestinal colonization with phylogenetic group B2 *Escherichia coli* related to inflammatory bowel disease: a systematic review and meta-analysis. *Scand. J. Gastroenterol.* 50 1199–1207. 10.3109/00365521.2015.1028993 25910859

[B133] Prey S. Paul C. Bronsard V. Puzenat E. Gourraud P. A. Aractingi S. (2010). Assessment of risk of psoriatic arthritis in patients with plaque psoriasis: a systematic review of the literature. *J. Eur. Acad. Dermatol. Venereol.* 24 31–35. 10.1111/j.1468-3083.2009.03565.x 20443998

[B134] Qin J. Li R. Raes J. Arumugam M. Burgdorf K. S. Manichanh C. (2010). A human gut microbial gene catalogue established by metagenomic sequencing: commentary. *Inflamm. Bowel Dis. Monit.* 11:28 10.1038/nature08821 PMC3779803 20203603

[B135] Qiu X. Zhang M. Yang X. Hong N. Yu C. (2013). *Faecalibacterium prausnitzii* upregulates regulatory T cells and anti-inflammatory cytokines in treating TNBS-induced colitis. *J. Crohn’s Colitis* 7 e558–e568. 10.1016/j.crohns.2013.04.002 23643066

[B136] Quera R. Espinoza R. Estay C. Rivera D. (2014). Bacteremia as an adverse event of fecal microbiota transplantation in a patient with Crohn’s disease and recurrent Clostridium difficile infection. *J. Crohns. Colitis* 8 252–253. 10.1016/j.crohns.2013.10.002 24184170

[B137] Rath H. C. Herfarth H. H. Ikeda J. S. Grenther W. B. Hamm T. E. Balish E. (1996). Normal luminal bacteria, especially bacteroides species, mediate chronic colitis, gastritis, and arthritis in HLA-B27/human B2 microglobulin transgenic rats. *J. Clin. Invest.* 98 945–953. 10.1172/JCI118878 8770866 PMC507509

[B138] Rehakova Z. Capkova J. Stepankova R. Sinkora J. Louzecka A. Ivanyi P. (2000). Germ-free mice do not develop ankylosing enthesopathy, a spontaneous joint disease. *Hum. Immunol.* 61 555–558. 10.1016/S0198-8859(00)00122-1 10825583

[B139] Rehaume L. M. Mondot S. Aguirre De Cárcer D. Velasco J. Benham H. Hasnain S. Z. (2014). ZAP-70 genotype disrupts the relationship between microbiota and host, leading to spondyloarthritis and ileitis in SKG mice. *Arthritis Rheumatol.* 66 2780–2792. 10.1002/art.38773 25048686

[B140] Rezende R. M. Oliveira R. P. Medeiros S. R. Gomes-Santos A. C. Alves A. C. Loli F. G. (2013). Hsp65-producing *Lactococcus lactis* prevents experimental autoimmune encephalomyelitis in mice by inducing CD4+LAP+ regulatory T cells. *J. Autoimmun.* 40 45–57. 10.1016/j.jaut.2012.07.012 22939403 PMC3623677

[B141] Rojo D. Hevia A. Bargiela R. López P. Cuervo A. González S. (2015). Ranking the impact of human health disorders on gut metabolism: systemic lupus erythematosus and obesity as study cases. *Sci. Rep.* 5:8310 10.1038/srep08310 PMC4319156 25655524

[B142] Round J. L. Mazmanian S. K. (2010). Inducible Foxp3+ regulatory T-cell development by∖na commensal bacterium of the intestinal microbiota. *Proc. Natl. Acad. Sci. U.S.A.* 107 12204–12209. 10.1073/pnas.0909122107 20566854 PMC2901479

[B143] Russell R. K. Ip B. Aldhous M. C. MacDougall M. Drummond H. E. Arnott I. D. R. (2009). Anti-Saccharomyces cerevisiae antibodies status is associated with oral involvement and disease severity in Crohn disease. *J. Pediatr. Gastroenterol. Nutr.* 48 161–167. 10.1097/MPG.0b013e318183e112 19179877

[B144] Ruutu M. Thomas G. Steck R. Degli-Esposti M. A. Zinkernagel M. S. Alexander K. (2012). B-Glucan triggers spondylarthritis and Crohn’s disease-like ileitis in SKG mice. *Arthritis Rheum.* 64 2211–2222. 10.1002/art.34423 22328069

[B145] Saviola G. Abdi-Ali L. Campostrini L. Sacco S. Baiardi P. Manfredi M. (2013). Clarithromycin in rheumatoid arthritis: the addition to methotrexate and low-dose methylprednisolone induces a significant additive value - A 24-month single-blind pilot study. *Rheumatol. Int.* 33 2833–2838. 10.1007/s00296-013-2822-0 23864141

[B146] Saviola G. Ali L. A. Rossini P. Campostrini L. Coppini A. Gori M. (2002). Clarithromycin in rheumatoid arthritis patients not responsive to disease-modifying antirheumatic drugs: an open, uncontrolled pilot study. *Clin. Exp. Rheumatol.* 20 373–378. 12102474

[B147] Scher J. U. Ubeda C. Artacho A. Attur M. Isaac S. Reddy S. M. (2015). Decreased bacterial diversity characterizes the altered gut microbiota in patients with psoriatic arthritis, resembling dysbiosis in inflammatory bowel disease. *Arthritis Rheumatol.* 67 128–139. 10.1002/art.38892 25319745 PMC4280348

[B148] Schlosstein L. Terasaki P. Bluestone R. Pearson C. (1973). High association of an HL-a antigen, W27, with ankylosing spondylitis. *N. Engl. J. Med.* 288 704–706. 10.1056/NEJM197304052881403 4688372

[B149] Shen J. Zuo Z. X. Mao A. P. (2014). Effect of probiotics on inducing remission and maintaining therapy in ulcerative colitis, Crohn’s disease, and pouchitis: meta-analysis of randomized controlled trials. *Inflamm. Bowel Dis.* 20 21–35. 10.1097/01.MIB.0000437495.30052.be 24280877

[B150] Sher J. Sczesnak A. Longman R. Segata N. Ubeda C. Bielski C. (2013). Expansion of intestinal Prevotella copri correlates with enhanced susceptibility to arthritis. *Elife* 2:e01202 10.7554/eLife.01202 PMC3816614 24192039

[B151] Shreiner A. B. Kao J. Y. Young V. B. (2015). The gut microbiome in health and disease. *Curr. Opin. Gastroenterol.* 31 69–75. 10.1053/j.gastro.2014.03.032 25394236 PMC4290017

[B152] Singh N. Gurav A. Sivaprakasam S. Brady E. Padia R. Shi H. (2014). Activation of Gpr109a, receptor for niacin and the commensal metabolite butyrate, suppresses colonic inflammation and carcinogenesis. *Immunity* 40 128–139. 10.1016/j.immuni.2013.12.007 24412617 PMC4305274

[B153] Sinkorova Z. Capkova J. Neiderlova J. Stepankova R. Sinkora J. (2008). Commensal intestinal bacterial strains trigger ankylosing enthesopathy of the ankle in inbred B10.BR (H-2k) male mice. *Hum. Immunol.* 69 845–850. 10.1016/j.humimm.2008.08.296 18840492

[B154] Smith P. M. Howitt M. R. Panikov N. Michaud M. Gallini C. A. Bohlooly-y M. (2013). The microbial metabolites, short-chain fatty acids, regulate colonic T cell homeostasis. *Science* 341 569–573. 10.1126/science.1241165 23828891 PMC3807819

[B155] Sokol H. Leducq V. Aschard H. Pham H. Jegou S. Landman C. (2016). Fungal microbiota dysbiosis in IBD. *Gut* 10.1136/gutjnl-2015-310746 [Epub ahead of print]. PMC5532459 26843508

[B156] Sokol H. Pigneur B. Watterlot L. Lakhdari O. Bermúdez-Humarán L. G. Gratadoux J.-J. (2008). *Faecalibacterium prausnitzii* is an anti-inflammatory commensal bacterium identified by gut microbiota analysis of Crohn disease patients. *Proc. Natl. Acad. Sci. U.S.A.* 105 16731–16736. 10.1073/pnas.0804812105 18936492 PMC2575488

[B157] Somers E. C. Thomas S. L. Smeeth L. Hall A. J. (2006). Autoimmune diseases co-occurring within individuals and within families. *Epidemiology* 17 202–217. 10.1097/01.ede.0000193605.93416.df 16477262

[B158] Sorrentino R. (2014). Genetics of autoimmunity: an update. *Immunol. Lett.* 158 116–119. 10.1016/j.imlet.2013.12.005 24370643

[B159] Stebbings S. Munro K. Simon M. A. Tannock G. Highton J. Harmsen H. (2002). Comparison of the faecal microflora of patients with ankylosing spondylitis and controls using molecular methods of analysis. *Rheumatology (Oxford)* 41 1395–1401. 10.1093/rheumatology/41.12.1395 12468819

[B160] Strauss J. Kaplan G. G. Beck P. L. Rioux K. Panaccione R. Devinney R. (2011). Invasive potential of gut mucosa-derived *Fusobacterium nucleatum* positively correlates with IBD status of the host. *Inflamm. Bowel Dis.* 17 1971–1978. 10.1002/ibd.21606 21830275

[B161] Svartz N. (1948). The treatment of rheumatic polyarthritis with acid azo compounds. *Rheumatism* 4 180–185. 18915673

[B162] Tap J. Mondot S. Levenez F. Pelletier E. Caron C. Furet J.-P. (2009). Towards the human intestinal microbiota phylogenetic core. *Environ. Microbiol.* 11 2574–2584. 10.1111/j.1462-2920.2009.01982.x 19601958

[B163] Taurog J. D. Richardson J. A. Croft J. T. Simmons W. A. Zhou M. Fernández-Sueiro J. L. (1994). The germfree state prevents development of gut and joint inflammatory disease in HLA-B27 transgenic rats. *J. Exp. Med.* 180 2359–2364. 10.1084/jem.180.6.2359 7964509 PMC2191772

[B164] Thomas G. P. Brown M. A. (2010). Genetics and genomics of ankylosing spondylitis. *Immunol. Rev.* 233 162–180. 10.1111/j.0105-2896.2009.00852.x 20192999

[B165] Tilley B. C. Alarcon G. S. Heyse S. P. Trentham D. E. Neuner R. Kaplan D. A. (1995). Minocycline in rheumatoid arthritis: a 48-week, double-blind, placebo-controlled trial. *Ann. Intern. Med.* 122 81–89. 10.7326/0003-4819-122-2-199501150-00001 7993000

[B166] Tremlett H. Fadrosh D. Farqui A. Hart J. Roalstad S. Graves J. (2016a). Associations between immune markers and gut microbiota in pediatric multiple sclerosis and controls (S29.008). *Neurology* 86:S29.008. 10.1186/s12883-016-0703-3 PMC5031272 27652609

[B167] Tremlett H. Fadrosh D. Lynch S. Hart K. Graves J. Lulu S. (2015). Gut microbiome in early pediatric multiple sclerosis: a case-control study (P4.027). *Neurology* 84.

[B168] Tremlett H. Fadrosh D. W. Faruqi A. A. Hart J. Roalstad S. Graves J. (2016b). Gut microbiota composition and relapse risk in pediatric MS: a pilot study. *J. Neurol. Sci.* 363 153–157. 10.1016/j.jns.2016.02.042 27000242 PMC4806409

[B169] Vaahtovuo J. Munukka E. Korkeamaki M. Luukkainen R. Toivanen P. (2008). Fecal microbiota in early rheumatoid arthritis. *J. Rheumatol.* 35 1500–1505. 18528968

[B170] Vaghef-Mehrabany E. Alipour B. Homayouni-Rad A. Sharif S.-K. K. Asghari-Jafarabadi M. Zavvari S. (2014). Probiotic supplementation improves inflammatory status in patients with rheumatoid arthritis. *Nutrition* 30 430–435. 10.1016/j.nut.2013.09.007 24355439

[B171] Valdimarsson H. Baker B. S. Jonsdottir I. Powles A. Fry L. (1995). Psoriasis: a T-cell-mediated autoimmune disease induced by streptococcal superantigens? *Immunol. Today* 16 145–149. 10.1016/0167-5699(95)80132-4 7718088

[B172] van den Broek M. F. van Bruggen M. C. Koopman J. P. Hazenberg M. P. van den Berg W. B. (1992). Gut flora induces and maintains resistance against streptococcal cell wall-induced arthritis in F344 rats. *Clin. Exp. Immunol.* 88 313–317. 10.1111/j.1365-2249.1992.tb03079.x 1572097 PMC1554307

[B173] van der Meulen T. A. Harmsen H. J. M. Bootsma H. Spijkervet F. K. L. Kroese F. G. M. Vissink A. (2016). The microbiome-systemic diseases connection. *Oral Dis.* 10.1111/odi.12472 [Epub ahead of print]. 26953630

[B174] van Nood E. Vrieze A. Nieuwdorp M. Fuentes S. Zoetendal E. G. de Vos W. M. (2013). Duodenal infusion of donor feces for recurrent *Clostridium difficile*. *N. Engl. J. Med.* 368 407–415. 10.1056/NEJMoa1205037 23323867

[B175] Vanaclocha F. Crespo-Erchiga V. Jiménez-Puya R. Puig L. Sánchez-Carazo J. L. Ferrán M. (2015). Immune-mediated inflammatory diseases and other comorbidities in patients with psoriasis: baseline characteristics of patients in the AQUILES study. *Actas Dermosifiliogr.* 106 35–43. 10.1016/j.ad.2014.06.003 25091923

[B176] Wagner J. Maksimovic J. Farries G. Sim W. H. Bishop R. F. Cameron D. J. (2013). Bacteriophages in gut samples from pediatric Crohn’s disease patients. *Inflamm. Bowel Dis.* 19 1598–1608. 10.1097/MIB.0b013e318292477c 23749273

[B177] Wang Y. Kasper L. H. (2014). The role of microbiome in central nervous system disorders. *Brain. Behav. Immun.* 38 1–12. 10.1016/j.bbi.2013.12.015 24370461 PMC4062078

[B178] Weng X. Liu L. Barcellos L. F. Allison J. E. Herrinton L. J. (2007). Clustering of inflammatory bowel disease with immune mediated diseases among members of a northern California-managed care organization. *Am. J. Gastroenterol.* 102 1429–1435. 10.1111/j.1572-0241.2007.01215.x 17437504

[B179] Wexler H. M. (2007). Bacteroides: the good, the bad, and the nitty-gritty. *Clin. Microbiol. Rev.* 20 593–621. 10.1128/CMR.00008-07 17934076 PMC2176045

[B180] Wu H. J. Ivanov I. I. Darce J. Hattori K. Shima T. Umesaki Y. (2010). Gut-residing segmented filamentous bacteria drive autoimmune arthritis via T helper 17 cells. *Immunity* 32 815–827. 10.1016/j.immuni.2010.06.001 20620945 PMC2904693

[B181] Wu X. Chen H. Xu H. (2015). The genomic landscape of human immune-mediated diseases. *J. Hum. Genet.* 60 675–681. 10.1038/jhg.2015.99 26290150

[B182] Zhang B. J. Markovic-plese S. Lacet B. Raus J. Weiner H. L. Hailer D. A. (1994). Increased frequency of interleukin 2-responsive T cells specific for myelin basic protein and proteolipid protein in peripheral blood and cerebrospinal fluid of patients with multiple sclerosis. *J. Exp. Med* 179 973–984. 10.1084/jem.179.3.973 7509366 PMC2191414

[B183] Zhang H. Liao X. Sparks J. B. Luo X. M. (2014). Dynamics of gut microbiota in autoimmune lupus. *Appl. Environ. Microbiol.* 80 7551–7560. 10.1128/AEM.02676-14 25261516 PMC4249226

[B184] Zhang X. Zhang D. Jia H. Feng Q. Wang D. Liang D. (2015). The oral and gut microbiomes are perturbed in rheumatoid arthritis and partly normalized after treatment. *Nat. Med.* 21 895–905. 10.1038/nm.3914 26214836

[B185] Zitomersky N. L. Atkinson B. J. Franklin S. W. Mitchell P. D. Snapper S. B. Comstock L. E. (2013). Characterization of adherent bacteroidales from intestinal biopsies of children and young adults with inflammatory bowel disease. *PLoS ONE* 8:e63686 10.1371/journal.pone.0063686 PMC3679120 23776434

